# Macrocycle with Equatorial Coordination Sites Provides New Opportunity for Structure-Diverse Metallacages

**DOI:** 10.3390/molecules28062537

**Published:** 2023-03-10

**Authors:** Yibo Zhao, Yunfeng Lu, Ao Liu, Zhi-Yuan Zhang, Chunju Li, Andrew C.-H. Sue

**Affiliations:** 1 School of Pharmaceutical Science and Technology, Tianjin University, Tianjin 300387, China; 2 Tianjin Key Laboratory of Structure and Performance for Functional Molecules, College of Chemistry, Tianjin Normal University, Tianjin 300387, China

**Keywords:** macrocyclic compounds, molecular cages, biphen[n]arenes, coordination-driven self-assembly, synthetic receptors, supramolecular chemistry

## Abstract

Reported here is the synthesis of a macrocycle with equatorial coordination sites for the construction of self-assembled metallacages. The macrocycle is prepared *via* a post-modification on the equator of biphen[*n*]arene. Utilizing this macrocycle as a ligand, three prismatic cages and one octahedral cage were synthesized by regulating the geometric structures and coordination number of metal acceptors. The multi-cavity configuration of prismatic cage was revealed by single-crystal structure. We prove that a macrocycle with equatorial coordination sites can be an excellent building block for synthesizing structure-diverse metallacages. Our results provide a typical example and a general method for the design and synthesis of metallacages.

## 1. Introduction

Molecular cages are widely utilized in catalysis, drug delivery, smart materials, chemical sensors, etc., due to their diverse building blocks, various geometry, and rich inner cavities [1,2,3,4,5,6]. Rebek and coworkers employed a cylindrical capsule as a nanoscale container for the 1,3-dipolar cycloaddition reaction between phenylazide and phenylacetylene. The cylindrical cavity of the capsule constrains the guests to be arranged in an edge-to-edge manner, resulting in the exclusive formation of the 1,4-triazole product after several days. Tiefenbacher et al. reported the application of an organic molecular cage as a catalyst for the selective hydrolysis of acetal derivatives in organic solvents. Mukherjee et al. demonstrated the utility of a trifacial molecular barrel-type molecular cage as a homogeneous catalyst in the efficient synthesis of xanthenes and their derivatives in aqueous media. Xu and colleagues employed an organic cage as a template to facilitate the synthesis of metal nanoparticles. In 2014, Zhang and coworkers presented a novel approach for the synthesis of gold nanoparticles (AuNPs) with controlled sizes based on a specific covalent organic cage. Therrien et al. demonstrated the formation of triangular prismatic host–guest compounds by self-assembling 2,4,6-tris(pyridin-4-yl)-1,3,5-triazine triangular panels with *p*-cymene ruthenium building blocks and 2,5-dioxydo-1,4-benzoquinonato bridges in the presence of a pyrenyl derivative (pyrene-R) that was functionalized. Nitschke et al. reported a fascinating collection of aromatic-paneled Fe_4_L_6_ cages created through iron(II)-templated subcomponent self-assembly of 2-formylpyridine and C_2_-symmetric diamine building blocks. The researchers discovered that both the size and the arrangement of the aromatic panels played a critical role in achieving successful encapsulation of large hydrophobic guests, such as fullerenes, polycyclic aromatic hydrocarbons, and steroids. They found that even minor differences in the structure of the subcomponents had obvious effects on the binding abilities of the resulting hosts.

Coordination-driven self-assembly is a simple and efficient method for constructing molecular cages [7,8,9,10,11,12,13]. A series of discrete or consecutive self-assembled metallacages with precisely controllable shapes and sizes (tetrahedron, triangular prisms, and octahedron) have been synthesized by this method [14,15,16]. Fujita and colleagues reported the use of a molecular cage as a nanoreactor for highly stable arenes (including pyrene, triphenylene, phenanthrene, fluoranthene, and perylene) that underwent an intermolecular [2 + 2] photoaddition reaction with N-cyclohexylmaleimide within the nanocavity of this cage. Sun et al. synthesized a water-soluble supramolecular cage by four palladium ions and two ligands. This cage exhibits redox activity and is able to encapsulate aromatic molecules and polyoxometalate catalysts. The well-defined cage possessed a high degree of stability and selectivity. Yan et al. found that the introduction of conformational restrictions on tetraphenylethene units had a significant impact on the structural relaxation in the excited state, as well as on the photophysical behaviors. Wurthner et al. reported a directional bonding approach involving assembling octahedral Fe(II) ions and linear perylene bisimide dyes with 2,2′-bipyridine groups by covalent connecting at the imide positions, resulting in the quantitative formation of a sizeable Fe_4_(PBI)_6_ tetrahedron. This remarkable structure had an impressive estimated internal volume of over 950 Å^3^ and an edge length of 3.9 nm. Jin et al. devised metallacages with cooperative dihydrogen binding sites for highly selectively capture cyclohexane molecules [17]. Generally, metallacages consist of appropriate electron donor ligands and electron-deficient metal-centered acceptors. Most properties of metallacages, such as luminescence, host–guest recognition, and biological toxicity, mainly depend on ligands [18,19,20,21,22]. Therefore, the selection of ligands is a crucial factor in determining the structure, properties, and applications of metallacages. Typically, small molecules are preferred as ligands because of their simple structures and convenient synthetic routes.

**Figure 1:**
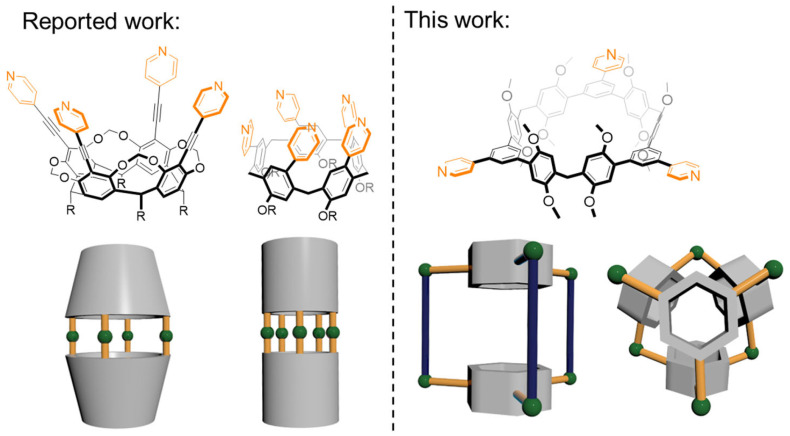
Schematic illustration of macrocyclic ligands with portal coordination sites (reported work) and equatorial coordination sites (this work) for the construction of coordination-driven self-assembly metallacages.

In addition to small molecules, macrocyclic hosts have also been used as ligands for metallacages [23,24]. The introduction of macrocycles to metallacages would provide interesting applications such as selective recognition, multi-guest molecule encapsulation, and molecular machines, due to the binding capacity of macrocycle cavities [25,26,27,28]. Therefore, the integration of macrocyclic ligands into metallacages opens up new approaches for the development of advanced materials with tailored properties and applications. Classic macrocyclic arenes such as calixarenes, resorcinarene, and pillararenes, where coordination sites can be easily introduced on their portals, are suitable for synthesizing capsule-like cages. Harrison et al. successfully synthesized a cavitand molecule with four iminodiacetate groups, which was subsequently self-assembled with cobalt(II) or iron(II) ions in water to form octa-anionic capsule molecules. The cobalt capsule has the ability to encapsulate a wide variety of guest molecules, including hydrocarbons, haloalkanes, aromatic compounds, alcohols, and ketones. This discovery showed various potential applications in molecular recognition and selective catalysis. Beer et al. reported a self-assembly of dithiocarbamate-functionalized cavitand molecules with late transition metals including Ni, Pd, Cu, Au, Zn, and Cd. This work expanded the previous findings by demonstrating the self-assembly of functionalized cavitands with a wider range of transition metals, thus opening up new possibilities for the design of complex and functional architectures. Based on a self-recognizable terpyridine-based ligand and Cd(II) ion system, Chan et al. reported a spontaneous heteroleptic complexation strategy. Utilizing this complementary ligand pairing approach, they successfully synthesized three types of nanocapsules, including a dimeric capsule, a Sierpiński triangular prism, and a cubic star, via dynamic complexation reactions between a tetratopic cavitand-based ligand and various multitopic counterparts in the presence of Cd(II) ions. The dimeric capsular assemblies exhibited spacer-length-dependent self-sorting behavior in a four-component system. Furthermore, the precise multicomponent self-assembly of a Sierpiński triangular prism and a cubic star, possessing three and six cavitand-based motifs, respectively, exhibited the potential of this self-assembly methodology for enhancing the architectural complexity of calix[4]resorcinarene-containing metallo-supramolecules. Martinez-Belmonte et al. prepared a novel class of self-assembled cages by conical-shaped carboxylic acid derivatives of calix[4]arene and calix[5]arene ligands and the metallic counterpart of uranyl cation. These cages exhibited hexagonal bipyramidal architectures, resulting from coordination between the uranyl cation and three carboxylate groups located in the equatorial plane. Sue et al. reported Ag_n_L_2_ metal–organic pillars assembled from pillar[5]arene, which paved the way for the construction of deep-cavity metallocavitands and nanochannels with unique molecular recognition and transportation properties [29,30,31] (Figure 1). We envision that macrocycles bearing equatorial coordination sites would provide a new opportunity for structure-diverse metallacages. However, the introduction of equatorial coordination sites into traditional macrocycles is usually difficult. Firstly, the modification on the skeleton is prohibitive for most macrocyclic compounds; secondly, the stereochemical structures of most macrocycles make it impossible to produce a derivate with equatorial coordination sites [32,33,34,35,36,37]. Therefore, the design and synthesis of macrocycle ligands with equatorial coordination sites remain a formidable challenge.

Herein, we report the synthesis of a macrocyclic compound containing pyridine groups at the equatorial plane for the construction of self-assembled metallacages. As far as we know, this is the first report of synthesizing metallacages from macrocyclic ligands bearing equatorial coordination sites.

## 2. Results and Discussion

**Scheme 1:**
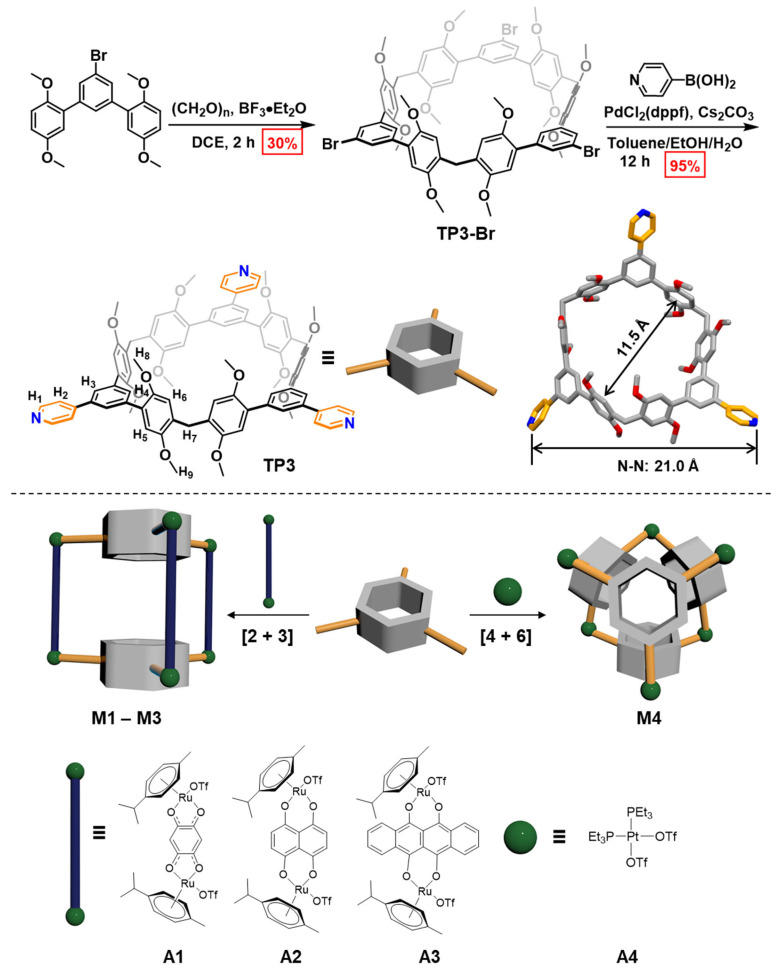
The synthesis and crystal structure of macrocyclic ligand (**TP3**) with equatorial coordination sites (**top**). **TP3** has a regular hexagonal cavity with a diameter of ~11.5 Å (C–C distance) and pyridine distance of ~21.0 Å (N–N distance). Cartoon representations of the assembly of [2 + 3] prismatic cages [4 + 6] and octahedron cage by macrocyclic ligand **TP3** and metal acceptors Ru(II) and Pt(II) (**bottom**).

In our previous work, we developed a modular synthetic strategy for functional macrocycles, which realizes the customization of size, functional backbones, and *endo*-binding sites of macrocycles [38,39,40]. This strategy endows macrocycles with potential applications in gas chromatography, pollutant capture, and physical adsorption separation [41,42]. Benefiting from the modular synthesis and plentiful molecular supplies, we readily synthesized the macrocyclic compound possessing pyridine groups on the outer side of the cavity. As illustrated in Scheme 1, the synthesis process of macrocyclic ligand **TP3** can be divided into three steps: firstly, the functional module tribromobenzene is coupled with the 2,5-dimethoxyphenyl reaction module via a Pd-catalyzed Suzuki–Miyaura cross-coupling reaction to obtain the monomer; secondly, the monomer is condensed with polyformaldehyde under the catalysis of Lewis acid boron trifluoride diethyl etherate to obtain **TP3-Br**; thirdly, the **TP3-Br** is coupled with pyridine boric acid to obtain the macrocycle **TP3**. Furthermore, the structure of **TP3** was unambiguously confirmed by the single-crystal structure (Scheme 1). **TP3** has a regular hexagonal cavity with a diameter of ~11.5 Å (C–C distance). The pyridine groups are located at the equator of the macrocycle molecules with a distance of ~21.0 Å (N–N distance). It is worth noting that the stacking structure of **TP3** is not parallel (Figure S33). The N atom of pyridine group forms an intermolecular hydrogen bond with the H atom on the OMe group of adjacent macrocycles (C–H···N 2.9 Å and 3.7 Å).

Subsequently, macrocyclic ligand **TP3** was used as a building block for the construction of two types of metallacages with half-sandwich [Ru_2_(µ-*η*^4^-OO∩OO)(*η*^6^-*p*-cymene)_2_](OTf)_2_ (OO∩OO = 2,5-dioxido-1,4-benzoquinonato (**A1**), 5,8-dioxido-1,4-naphtoquinonato (**A2**), 6,11-dihydroxy-5,12-naphthacenedione (**A3**)), and *cis*-Pt-(PEt_3_)_2_(OTf)_2_ (**A4**) acceptors. As shown in Scheme 1, the prismatic cages **M1**–**M3** were constructed by mixing macrocyclic ligand **TP3** with binuclear Ru(II) acceptors **A1**–**A3** in CH_2_Cl_2_:CH_3_OH (*v*:*v* = 1:1) under stirring at room temperature for 12 h and then evaporating the solvents. Minor CH_3_OH was added to the dried residues, followed by the addition of diethyl ether. The resulting precipitates were collected by centrifugation. **M1**–**M3** were obtained in 83–86% yields. Following this method, the octahedral cage **M4** was constructed by stirring ligand **TP3** with Pt(II) acceptors **A4** in CH_2_Cl_2_:(CH_3_)_2_CO (*v*:*v* = 2:1) at room temperature for 24 h. **M4** was obtained in 80% yield. All the metallacages were isolated as triflate salts and are highly soluble in common organic solvents like CH_3_OH, CH_3_CN, (CH_3_)_2_CO, and CH_2_Cl_2_. The formation of metallacages **M1**–**M4** was supported by NMR spectrum (^1^H, ^31^P (just **M4**), ^13^C, COSY, DOSY, and NOESY) and electrospray ionization mass spectrometry (ESI-MS) analyses.

**Figure 2:**
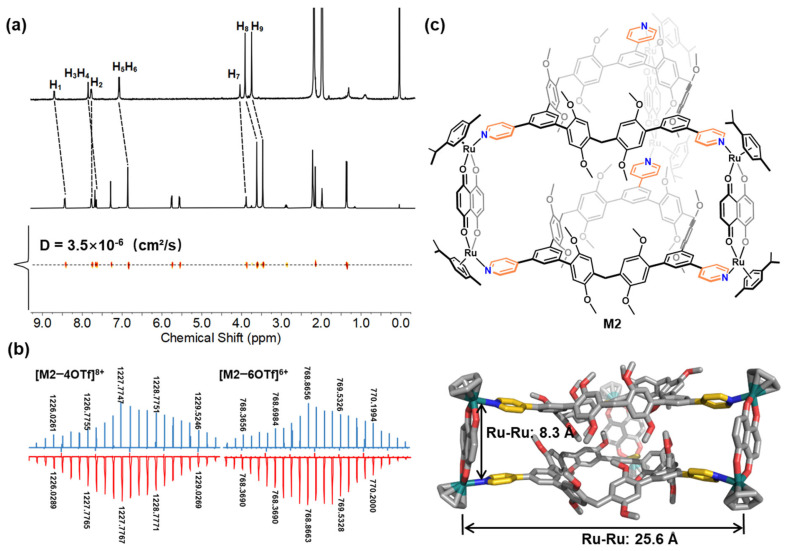
(**a**) ^1^H and DOSY NMR spectra of ligand **TP3** and metallacage **M2** in CD_3_CN. (**b**) Calculated (blue) and experimental (red) ESI-MS of **M2**. (**c**) Chemical structure and crystal structure of **M2** from the side views. The solvents, counterions, H atoms, and methyl/isopropyl in cymene are omitted for clarity. Color code: gray = C; red = O; blue = N; green = Ru; gold = pyridine units.

**Figure 3:**
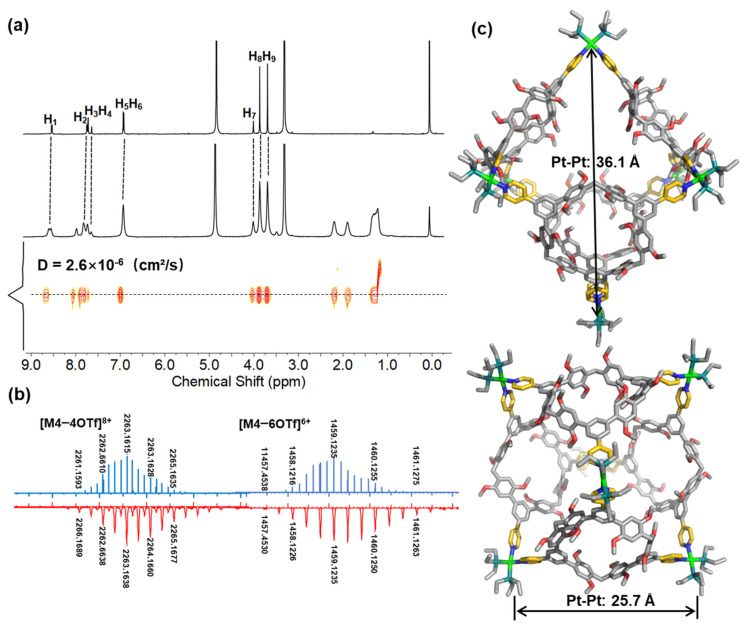
(**a**) ^1^H and DOSY NMR spectra of ligand **TP3** and metallacage **M4** in CD_3_OD. (**b**) Calculated (blue) and experimental (red) ESI-MS of **M4**. (**c**) Optimized structure of **M4** from the top and side views. The solvents, counterions, and H atoms are omitted for clarity. Color code: gray = C; red = O; blue = N; dark green = P; green = Pt; gold = pyridine units.

The self-assembly behavior between the acceptors **A1**–**A4** and the ligand **TP3** was carefully studied using NMR spectroscopy in CD_3_CN or CD_3_OD. The ^1^H NMR spectra of the resulting metallacages **M1**–**M4** revealed that they possess highly symmetric and discrete structures. Upon the formation of the metallacages, the partial proton signals of the ligand **TP3** displayed significant shifts, which can be attributed to the loss of electron density upon ligand-to-metal coordination (Figure 2a and Figure 3a). In addition, diffusion-ordered NMR spectroscopy (DOSY) analysis was conducted, which further confirmed the formation of the metallacages in solution (Figure 2a, Figure 3a, Figures S13 and S23). Specifically, in the DOSY spectra, **M1**–**M4** displayed a single band of signals with a diffusion coefficient (D) of 2.8 × 10^−6^ cm^2^/s for **M1** in CD_3_CN, 3.5 × 10^−6^ cm^2^/s for **M2** in CD_3_CN, 2.1 × 10^−6^ cm^2^/s for **M3** in CD_3_CN, and 2.6 × 10^−6^ cm^2^/s for **M4** in CD_3_OD at 298 K, indicating the presence of a single species of metallacages. These results provide strong evidence for the successful formation of the highly symmetric and discrete metallacages through self-assembly between the acceptors and the ligand **TP3**.

Further confirmation of the formation of the prismatic metallacages **M1**–**M3** and octahedral cage **M4** was obtained through ESI-MS studies. The ESI-MS spectrum of **M1**–**M3** displayed peaks corresponding to the assigned [2 + 3] assembly, including peaks with continuous charge states ranging from 3^+^ to 6^+^, resulting from the successive loss of the counteranion OTf^−^ (as shown in Figure 2b and Figures S29–S31). Similarly, the ESI-MS results for **M4** revealed peaks for the assigned [4 + 6] assembly (Figure 3b and Figure S32). All peaks in the mass spectra were isotopically resolved and agreed well with their calculated theoretical distributions. These results provide further evidence of the successful formation of the desired metallacages. For **M1**, peaks were observed corresponding to [**M1**–3OTf]^3+^ (*m*/*z* 1636.6801), [**M1**–4OTf]^4+^ (*m*/*z* 1190.2698), [**M1**–5OTf]^5+^ (*m*/*z* 922.4241), and [**M1**–6OTf]^6+^ (*m*/*z* 743.8596); for **M2**, peaks were observed corresponding to [**M2**–3Otf]^3+^ (*m*/*z* 1686.6885), [**M2**–4Otf]^4+^ (*m*/*z* 1227.7767), [**M2**–5Otf]^5+^ (*m*/*z* 952.4299), and [**M2**–6OTf]^6+^ (*m*/*z* 768.8663); for **M3**, peaks were observed corresponding to [**M3**–3OTf]^3+^ (*m*/*z* 1787.0522), [**M3**–4OTf]^4+^ (*m*/*z* 1303.0518), [**M3**–5OTf]^5+^ (*m*/*z* 1012.6496), and [**M3**–6OTf]^6+^ (*m*/*z* 819.0487); for **M4**, peaks were observed corresponding to [**M4**–4OTf]^8+^ (*m*/*z* 2263.1638), [**M4**–6OTf]^6+^ (*m*/*z* 1459.1241), and [**M4**–8OTf]^4+^ (*m*/*z* 1057.1041).

Single-crystal X-ray diffraction analysis further elucidated the structure of prismatic cage **M2**. The X-ray-quality single crystal of the self-assembly metallacage **M2** was obtained as atrovirens cube crystal by vapor diffusion of *i*-propyl ether into acetonitrile. As illustrated in Figure 2c, the solid-state structure of **M2** revealed that two pyridine-based macrocycle **TP3** were connected by three ruthenium(II) acceptors. Two macrocyclic molecules form the bottoms of the triangular prism. Interestingly, they are not parallel to each other, which may contribute to an increase in the C–H···π interactions (2.6, 2.8, and 3.0 Å). Some methoxy groups on the macrocyclic molecule inserted into the cavity of another macrocycle via multiple C–H···π interactions. The hydrophobic cavities of the upper and lower were arranged together to form a channel. The Ru(II) acceptor forms the three sides of the triangular prism. They are also not completely vertical. The side length of the bottom surface of the triangular prism is ~25.6 Å (Ru–Ru distance), and the height of the triangular prism is ~8.3 Å (Ru–Ru distance). For the packing structure, adjacent metallacage units are packed in a parallel stacking with a separation of ~5.6 Å (Figure S34); no C–H···π hydrogen bonds can be found.

Despite multiple attempts, it was not possible to obtain a single crystal of **M4**. Therefore, geometry optimizations were performed using density functional theory (DFT) calculations with the B3LYP/3-21G method. The results of these calculations revealed that **M4** has an explicit octahedral conformation. Specifically, the octahedral cage is formed by four macrocyclic ligands (**TP3**), which form the four faces of the cage, and six Pt(II) acceptors, which form the verticals. The side length of this octahedral cage is 25.7 Å, which is the distance between the Pt atoms. The simulated structure provides a detailed representation of **M4**’s geometry, which is important for understanding its properties and potential applications. Despite the inability to obtain a single crystal of **M4**, the DFT calculations provide valuable insights into its structural characteristics.

## 3. Materials and Methods

All the chemicals used in this study were purchased from commercial sources and were not subjected to any further purification. The nuclear magnetic resonance (NMR) spectra, including ^1^H, ^31^P (only for **M4**), ^13^C, COSY, DOSY, and NOESY, were recorded on a Bruker Avance 400/600 MHz spectrometer. The chemical shifts in the NMR spectra are reported in parts per million (ppm) relative to the proton resonance resulting from incomplete deuteration of the NMR solvents, which were CD_3_OD (3.33 ppm for ^1^H and 49.0 ppm for ^13^C), CD_3_CN (1.94 ppm for ^1^H and 118.3 ppm for ^13^C), and CDCl_3_ (7.26 ppm for ^1^H and 77.2 ppm for ^13^C). High-resolution electrospray ionization (HR-ESI) mass spectral analyses were performed using the Thermo Fisher Q Exactive™ HF/UltiMate™ 3000 RSLCnano. Matrix Assisted Laser Desorption Ionization (MALDI) mass spectra were performed on Bruker Daltonics UltrafleXtreme time of flight (TOF) equipment. Single crystals suitable for X-ray crystallographic analysis were selected, and their X-ray diffraction intensity data were collected on a rotating anode diffractometer equipped with a hybrid photon counting detector. Graphite-monochromated CuKα radiation with a wavelength of 1.54184 Å was used at a temperature of 200 K. To simulate the geometry optimizations of the metallacage **M4**, the Gaussian 09 program was used with B3LYP/3-21G computations. The obtained results provide valuable insights into the structure and properties of the metallacage **M4**, which are important for understanding its potential applications. The use of advanced techniques, such as HR-ESI mass spectrometry and X-ray crystallographic analysis, ensures the accuracy and reliability of the results, while the NMR spectra provide additional information about the chemical environment of the metallacages.

The acceptors [Ru_2_(µ-*η*^4^-OO∩OO)(*η*^6^-*p*-cymene)_2_](OTf)_2_ (OO∩OO = 2,5-dioxido-1,4-benzoquinonato (dobq) and 5,8-dioxido-1,4-naphtoquinonato (donq) and 6,11-dihydroxy-5,12-naphthacenedione (dhnc)) and *cis*-Pt-(PEt_3_)_2_(OTf)_2_ were synthesized under dry nitrogen atmosphere using standard Schlenk technique following the reported procedures [43,44,45,46].

5′-bromo-2,2″,5,5″-tetramethoxy-1,1′:3′,1″-terphenyl: A mixture of tribromobenzene (1.0 g, 3.2 mmol), 2,5-dimethoxybenzene boronic acid (1.28 g, 7.0 mmol), [1,1′-bis(diphenylphosphino)-ferrocene]dichloropalladium (0.12 g, 0.18 mmol), and K_2_CO_3_ (1.3 g, 9.6 mmol) was dissolved in 60 mL of a mixed solvent made up of dioxane and water (*v*/*v* = 5:1) in a flask. The mixture was stirred at 90 °C for 12 h in a nitrogen atmosphere. After cooling down to room temperature, the solvent was distilled under reduce pressure. The resulting mixture was extracted with CH_2_Cl_2_ (3 × 50 mL) and then washed with water and brine successively. The organic layer was dried over anhydrous Na_2_SO_4_ and concentrated. The residue was purified by column chromatography on silica gel (eluent petroleum ether: dichloromethane 2:1, *v*/*v*) to afford 5′-bromo-2,2″,5,5″-tetramethoxy-1,1′:3′,1″-terphenyl (0.8 g, 59%). ^1^H-NMR (400 MHz, CDCl_3_) δ = (ppm) 7.65 (d, *J* = 1.5 Hz, 2H), 7.61 (t, *J* = 1.5 Hz, 1H), 6.95-6.86 (m, 6H), 3.80 (s, 6H), 3.77 (s, 6H). ^13^C-NMR (100 MHz, CDCl_3_) δ = (ppm) 153.8, 150. 8, 140.0, 131.1, 130.2, 129.5, 121.6, 116.7, 113.8, 112.7, 56.4, 55.9.

**TP3-Br**: A solution of 5′-bromo-2,2″,5,5″-tetramethoxy-1,1′:3′,1″-terphenyl (0.50 g, 1.2 mmol) and paraformaldehyde (0.11 g, 3.6 mmol) in 1,2-dichloroethane (100 mL) was prepared, and boron trifluoride etherate (0.10 mL, 1.0 mmol) was added to the solution. The reaction mixture was then stirred at room temperature for 2 h. After completion of the reaction, the mixture was quenched by the addition of 100 mL of saturated NaHCO_3_ solution. The organic phase was then separated and washed with saturated NaHCO_3_ solution and brine. The organic layer was dried over anhydrous Na_2_SO_4_ and the solvent was removed by evaporation. The resultant residue was purified by column chromatography on silica gel using petroleum ether:dichloromethane (1:1.5, *v*/*v*) as the eluent to yield **TP3-Br** (0.47 g, 30%). ^1^H-NMR (400 MHz, CDCl_3_) δ = (ppm) 7.58 (d, *J* = 1.5 Hz, 6H), 7.57–7.56 (m, 3H), 6.85 (s, 6H), 6.83 (s, 6H), 4.01 (s, 6H), 3.84 (s, 18H), 3.66 (s, 18H). ^13^C-NMR (100 MHz, CDCl_3_) δ = (ppm) 151.8, 150.4, 140.2, 130.4, 130.4, 129.9, 127.8, 121.9, 115.0, 113.6, 56.4, 56.3, 30.3.

**TP3**: A mixture of **TP3-Br** (0.47 g, 0.35 mmol), pyridine-4-boronic acid (0.43 g, 3.5 mmol), [1,1′-bis(diphenylphosphino)ferrocene]dichloropalladium (0.02 g, 0.03 mmol), and Cs_2_CO_3_ (0.57 g, 1.75 mmol) were dissolved in 78 mL of a mixed solvent made up of toluene, ethanol and water (*v*/*v*/*v* = 8:4:1) in a flask. The mixture was stirred at 90 °C for 12 h in a nitrogen atmosphere. After cooling down to room temperature, the solvent was distilled under reduce pressure. Resulting mixture was extracted with CH_2_Cl_2_ (3 × 50 mL) and then washed with water and brine successively. The organic layer was dried over anhydrous Na_2_SO_4_ and concentrated. The residue was purified by column chromatography on silica gel (eluent dichloromethane: methanol 100:1, *v*/*v*) to afford **TP3** (0.44 g, 95%). ^1^H-NMR (600 MHz, CDCl_3_) δ = (ppm) 8.70 (s, 6H), 7.68 (d, *J* = 67.1 Hz, 15H), 6.94 (s, 12H), 4.07 (s, 6H), 3.88 (s, 18H), 3.71 (s, 18H). ^13^C-NMR (100 MHz, CDCl_3_) δ = (ppm) 151.9, 150.5, 150.2, 149.0, 139.3, 138.1, 132.4, 129.8, 128.7, 126.5, 122.2, 115.0, 113.8, 56.5, 56.4, 30.4. MALDI-TOF MS: C_84_H_75_N_3_O_12_, calculated for *m*/*z* 1317.535; found 1317.596.

General procedure for prismatic metallacages **M1**–**M3**: In a 2:3 molar ratio, the ligand **TP3** and acceptors [Ru_2_(µ-*η*^4^-OO∩OO)(*η*^6^-*p*-cymene)_2_]OTf_2_ (**A1**–**A3**) were placed in a 10 mL vial, followed by addition of CH_2_Cl_2_ (3 mL) and CH_3_OH (3 mL). After stirring at ambient temperature for 24 h, the solution was concentrated to 0.5 mL. The self-assembly products were isolated via precipitation by addition of diethyl ether into concentrated solution, washed twice with diethyl ether and dried under vacuum.

**M1**: Reaction scale: **A1** (9.2 mg, 0.01 mmol) and **TP3** (9.2 mg, 0.007 mmol). Compound **M1** was obtained as a red powder, 12.3 mg, 83% yield. ^1^H-NMR (400 MHz, CD_3_CN) δ = (ppm) 8.26 (s, 12H), 7.81 (s, 12H), 7.69 (s, 12H), 7.61 (s, 6H), 6.83 (s, 24H), 5.91 (s, 12H), 5.79 (s, 6H), 5.69 (s, 12H), 3.87 (s, 12H), 3.57 (s, 36H), 3.43 (s, 36H), 2.96–2.75 (m, 6H), 2.07 (s, 18H), 1.34 (d, *J* = 4.9 Hz, 36H). ^13^C-NMR (100 MHz, CD_3_CN) δ = (ppm) 185.2, 153.8, 152.6, 151.8, 150.4, 140.4, 135.7, 134.2, 130.3, 128.7, 127.4, 124.6, 115.7, 113.8, 104.5, 102.2, 99.1, 84.6, 82.7, 56.6, 56.5, 32.1, 31.1, 22.4, 18.2. ESI-MS: *m*/*z* calculated for [**M1**–4OTf]^8+^: 1190.2630; found 1190.2698.

**M2**: Reaction scale: **A2** (9.6 mg, 0.01 mmol) and **TP3** (9.2 mg, 0.007 mmol). Compound **M2** was obtained as a green powder, 13.1 mg, 85% yield. ^1^H-NMR (400 MHz, CD_3_CN) δ = (ppm) 8.38 (t, *J* = 6.3 Hz, 12H), 7.72 (d, *J* = 6.5 Hz, 12H), 7.63 (d, *J* = 1.1 Hz, 12H), 7.59 (d, *J* = 5.9 Hz, 6H), 7.23 (d, *J* = 7.0 Hz, 12H), 6.88-6.73 (m, 24H), 5.70 (t, *J* = 5.9 Hz, 12H), 5.50 (t, *J* = 5.9 Hz, 12H), 3.85 (d, *J* = 11.1 Hz, 12H), 3.57 (s, 36H), 3.44 (d, *J* = 16.4 Hz, 36H), 2.90–2.67 (m, 6H), 2.10 (d, *J* = 6.9 Hz, 18H), 1.31 (t, *J* = 7.5 Hz, 36H). ^13^C-NMR (100 MHz, CD_3_CN) δ = (ppm) 171.9, 153.0, 152.6, 151.9, 151.1, 140.3, 138.4, 136.1, 134.0, 130.3, 128.8, 127.4, 124.5, 115.8, 114.2, 112.4, 104.5, 100.2, 85.1, 84.0, 56.7, 56.5, 31.4, 31.1, 22.3, 17.3. ESI-MS: *m*/*z* calculated for [**M2**–4OTf]^8+^: 1227.7747; found 1227.7767.

**M3**: Reaction scale: **A3** (10.6 mg, 0.01 mmol) and **TP3** (9.2 mg, 0.007 mmol). Compound **M3** was obtained as a dark-blue powder, 14.1 mg, 86% yield. ^1^H-NMR (400 MHz, CD_3_OD) δ = (ppm) 8.78 (s, 12H), 8.57 (d, *J* = 5.4 Hz, 12H), 7.98 (s, 12H), 7.69 (d, *J* = 5.2 Hz, 12H), 7.54 (s, 12H), 7.42 (s, 6H), 6.66 (d, *J* = 5.1 Hz, 24H), 6.02 (d, *J* = 5.8 Hz, 12H), 5.78 (d, *J* = 5.8 Hz, 12H), 3.83 (dd, *J* = 24.4, 14.6 Hz, 12H), 3.57-3.47 (m, 36H), 3.31 (s, 36H), 3.01–2.93 (m, 6H), 2.23 (s, 18H), 1.34 (dd, *J* = 23.2, 6.6 Hz, 36H). ^13^C-NMR (100 MHz, CD_3_OD) δ = (ppm) 170.6, 153.2, 151.5, 140.7, 135.1, 134.3, 130.9, 129.7, 128.4, 127.2, 124.5, 116.3, 114.2, 104.9, 101.0, 85.1, 83.6, 66.9, 56.9, 56.5, 32.1, 22.6, 17.9, 15.4. ESI-MS: *m*/*z* calculated for [**M3**–4OTf]^8+^: 1303.0490; found 1303.0518.

**M4**: At a 4:6 molar ratio, the platinum complex **A4** (5.3 mg, 0.007 mmol) and **TP3** (6.4 mg, 0.005 mmol) were placed in a 10 mL vial, followed by addition of CH_2_Cl_2_ (3 mL) and (CH_3_)_2_CO (3 mL). After stirring at ambient temperature for 24 h, the solution was concentrated. The self-assembly products were isolated via precipitation by adding diethyl ether into the concentrated solution, washing twice with diethyl ether, and drying under vacuum to obtain product **M4** (20.1 mg, 60% yield). ^1^H-NMR (400 MHz, CD_3_OD) δ = (ppm) 8.64 (d, *J* = 14.1 Hz, 24H), 8.17–7.59 (m, 60H), 6.97 (s, 48H), 4.01 (s, 24H), 3.86 (s, 72H), 3.69 (s, 72H), 2.17 (s, 36H), 1.85 (s, 36H), 1.33–1.18 (m, 108H). ESI-MS: *m*/*z* calculated for [**M4**–4OTf]^8+^: 2263.1615; found 2263.1638.

## 4. Conclusions

In summary, we successfully synthesized a macrocycle **TP3** with pyridine units on the equator *via* a post-modification of biphenarene. Benefiting from the equatorial coordination sites, the macrocycle could be an excellent ligand for the construction of four novel prismatic/octahedral cages **M1**–**M4** *via* coordination-driven self-assembly with Ru(II) and Pt(II) building acceptors. All these metallacages were fully characterized by NMR and ESI-MS spectroscopic studies. Moreover, the multi-cavity configuration of the prismatic cage was proven by X-ray crystallographic analysis. This work enriches the toolbox of macrocycle ligands and provides a general method for the design and synthesis of structure-diverse metallacages. More macrocycles with equatorial coordination sites can be synthesized, various macrocycle-based metallacages can be assembled, and further functions and applications can be explored.

## Data Availability

Data can be made available upon reasonable request.

## Supplementary Materials

The following supporting information can be downloaded at https://www.mdpi.com/article/10.3390/molecules28062537/s1. Figure S1. 1H NMR spectrum (400 MHz, CDCl3, 298 K) of 5′-bromo-2,2″,5,5″-tetramethoxy-1,1′:3′,1″-terphenyl. Figure S2. 13C NMR spectrum (100 MHz, CDCl3, 298 K) of 5′-bromo-2,2″,5,5″-tetramethoxy-1,1′:3′,1″-terphenyl. Figure S3. 1H NMR spectrum (400 MHz, CDCl3, 298 K) of TP3-Br. Figure S4. 13C NMR spectrum (100 MHz, CDCl3, 298 K) of TP3-Br. Figure S5. 1H NMR spectrum (600 MHz, CDCl3, 298 K) of TP3. Figure S6. 13C NMR spectrum (100 MHz, CDCl3, 298 K) of TP3-Br. Figure S7. MALDI-TOF MS of TP3-Br. Figure S8. 2D COSY spectrum (400 MHz, CDCl3, 298 K) of TP3-Br. Figure S9. 1H NMR spectrum (400 MHz, CD3CN, 298 K) of M1. Figure S10. 13C NMR spectrum (100 MHz, CD3CN, 298 K) of M1. Figure S11. 2D COSY spectrum (400 MHz, CD3CN, 298 K) of M1. Figure S12. 2D NOESY spectrum (400 MHz, CD3CN, 298 K) of M1. Figure S13. DOSY spectrum (400 MHz, CD3CN, 298 K) of M1. Figure S14. 1H NMR spectrum (400 MHz, CD3CN, 298 K) of M2. Figure S15. 13C NMR spectrum (100 MHz, CD3CN, 298 K) of M2. Figure S16. 2D COSY spectrum (400 MHz, CD3CN, 298 K) of M2. Figure S17. 2D NOESY spectrum (400 MHz, CD3CN, 298 K) of M2. Figure S18. DOSY spectrum (400 MHz, CD3CN, 298 K) of M2. Scheme S3. Synthetic routes for the M3. Figure S19. 1H NMR spectrum (400 MHz, CD3OD, 298 K) of M3. Figure S20. 13C NMR spectrum (100 MHz, CD3OD, 298 K) of M3. Figure S21. 2D COSY spectrum (400 MHz, CD3OD, 298 K) of M3. Figure S22. 2D NOESY spectrum (400 MHz, CD3OD, 298 K) of M3. Figure S23. DOSY spectrum (400 MHz, CD3CN, 298 K) of M3. Figure S24. 1H NMR spectrum (400 MHz, CD3OD, 298 K) of M4. Figure S25. 31P{1H} NMR (162 MHz, CD3OD, 25 °C) spectrum of M4. Figure S26. 2D COSY spectrum (400 MHz, CD3OD, 298 K) of M4. Figure S27. 2D NOESY spectrum (400 MHz, CD3OD, 298 K) of M4. Figure S28. DOSY spectrum (400 MHz, CD3OD, 298 K) of M4. Figure S29. ESI-MS spectra of M1. Figure S30. ESI-MS spectra of M2. Figure S31. ESI-MS spectra of M3. Figure S32. ESI-MS spectra of M4. Figure S33. Packing diagram of TP3 view along the (a) a-axis, (b) b-axis, and (c) c-axis. Figure S34. Packing diagram of M2 view along the (a) a-axis, (b) b-axis, and (c) c-axis. Table S1. Crystal data of TP3. Table S2. Crystal data of M2. Scheme S1. Synthetic routes for the M1. Scheme S2. Synthetic routes for the M2.

## Sample Availability

Samples of the compounds are available from the authors upon reasonable request.
