# Supplementary material for: Macrocycle with Equatorial Coordination Sites Provides New Opportunity for Structure-Diverse Metallacages
Source: Molecules. 2023 Mar 10;28(6):2537. doi: 10.3390/molecules28062537 (PMC10059262; doi:10.3390/molecules28062537)
Supplement: Supplementary file 1 [file molecules-28-02537-s001.zip › molecules-2249344-supplementary.pdf]

Supporting Information

Macrocycle with Equatorial Coordination Sites Provides New Opportunity for Structure-Diverse Metallacages

Yibo Zhao [1], Yunfeng Lu [2], Ao Liu [2], Zhi-Yuan Zhang [2,*], Chunju Li [2,*] and Andrew C.-H. Sue [1,*]

[1] School of Pharmaceutical Science and Technology, Tianjin University, Tianjin 300387, China
[2] Tianjin Key Laboratory of Structure and Performance for Functional Molecules, College of Chemistry, Tianjin Normal University, Tianjin 300387, China
* Correspondence: zzy@tjnu.edu.cn (Z.-Y.Z.); cjli@shu.edu.cn (C.L.); andrewsue@xmu.edu.cn (A.C.-H.S.)

Table of Contents

Section I. Synthesis and characterization of self-assembled metallacages…S2-S22

Section II. Single-crystal structures of TP3 and M2……………………….S23-S26

Section I. Synthesis and characterization of self-assembled metallacages

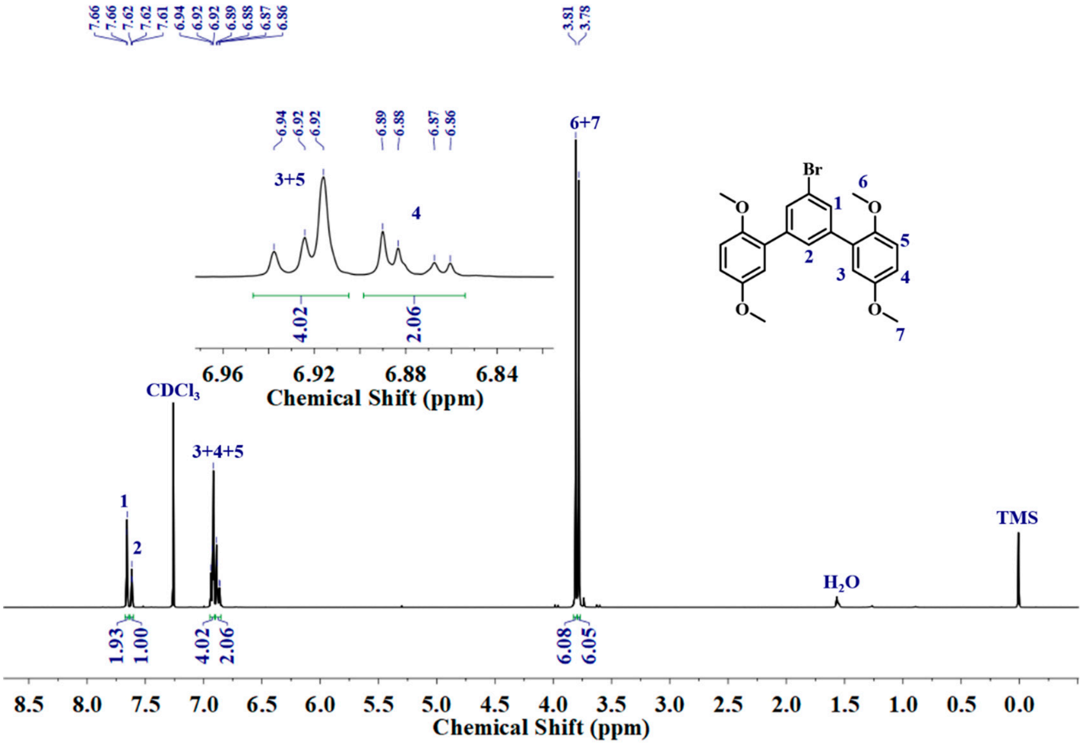

Figure S1. ^{1}H NMR spectrum (400 MHz, $CDCl_3$, 298 K) of 5'-bromo-2,2",5,5"-tetramethoxy-1,1':3',1"-terphenyl.

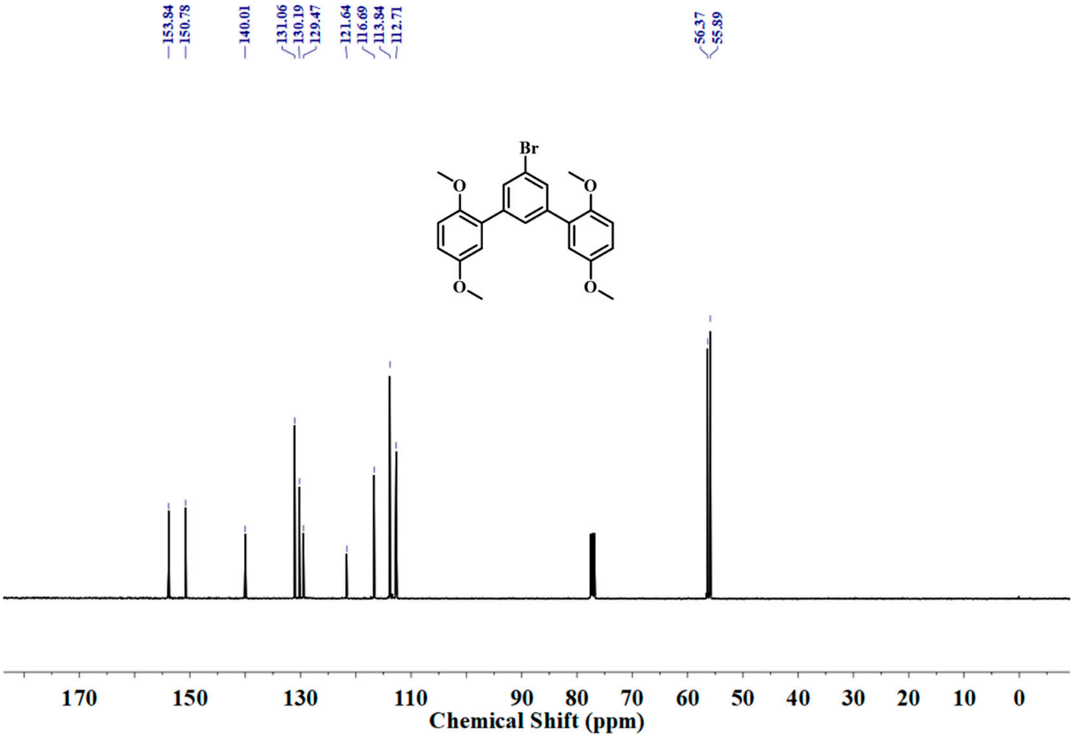

Figure S2. ^{13}C NMR spectrum (100 MHz, $CDCl_3$, 298 K) of 5'-bromo-2,2",5,5"-tetramethoxy-1,1':3',1"-terphenyl.

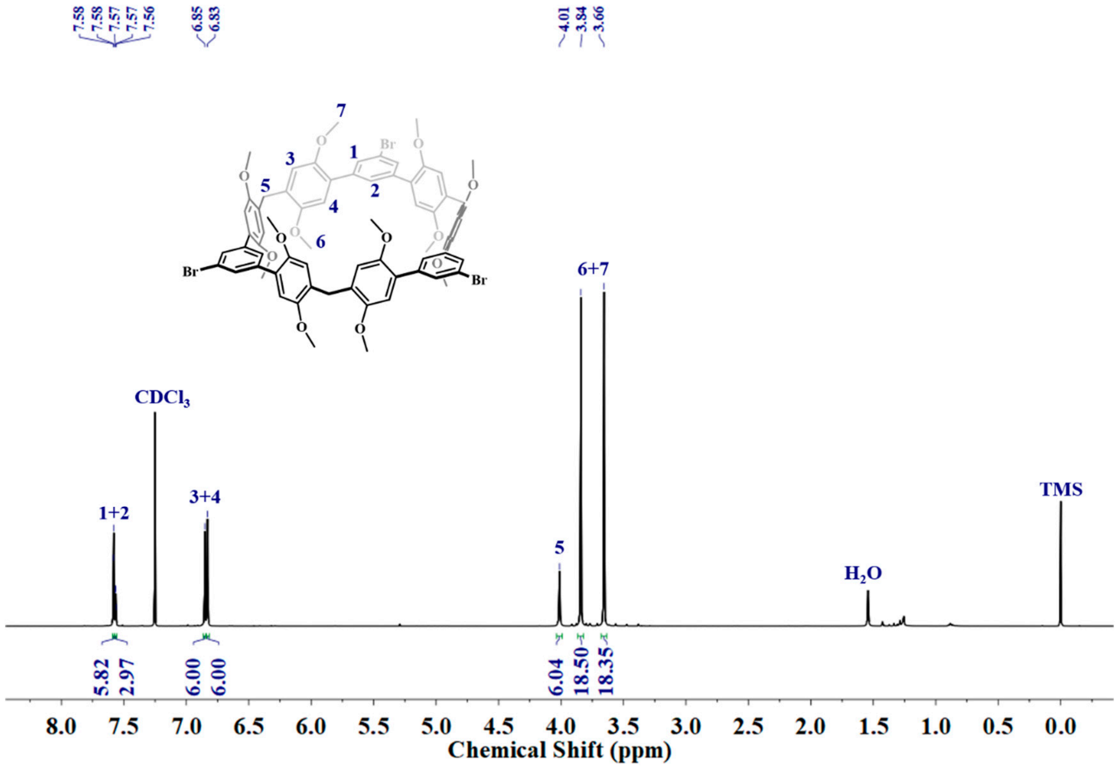

Figure S3. ^{1}H NMR spectrum (400 MHz, $CDCl_3$, 298 K) of **TP3-Br**.

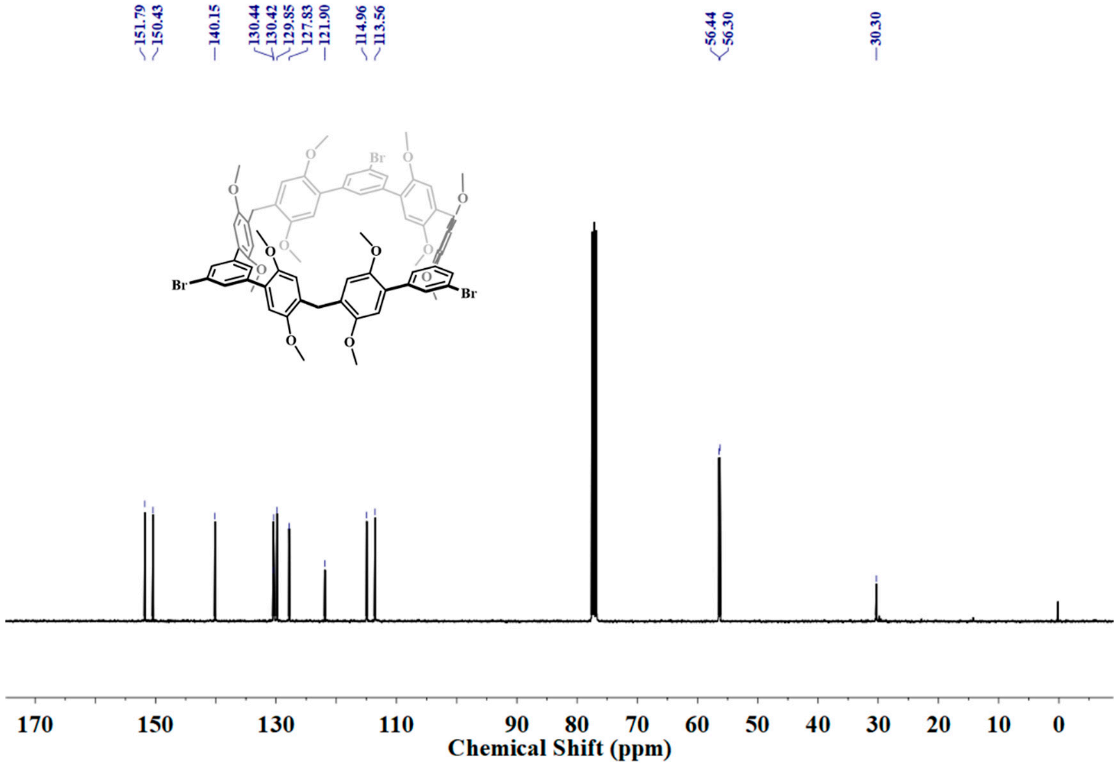

Figure S4. ^{13}C NMR spectrum (100 MHz, $CDCl_3$, 298 K) of **TP3-Br**.

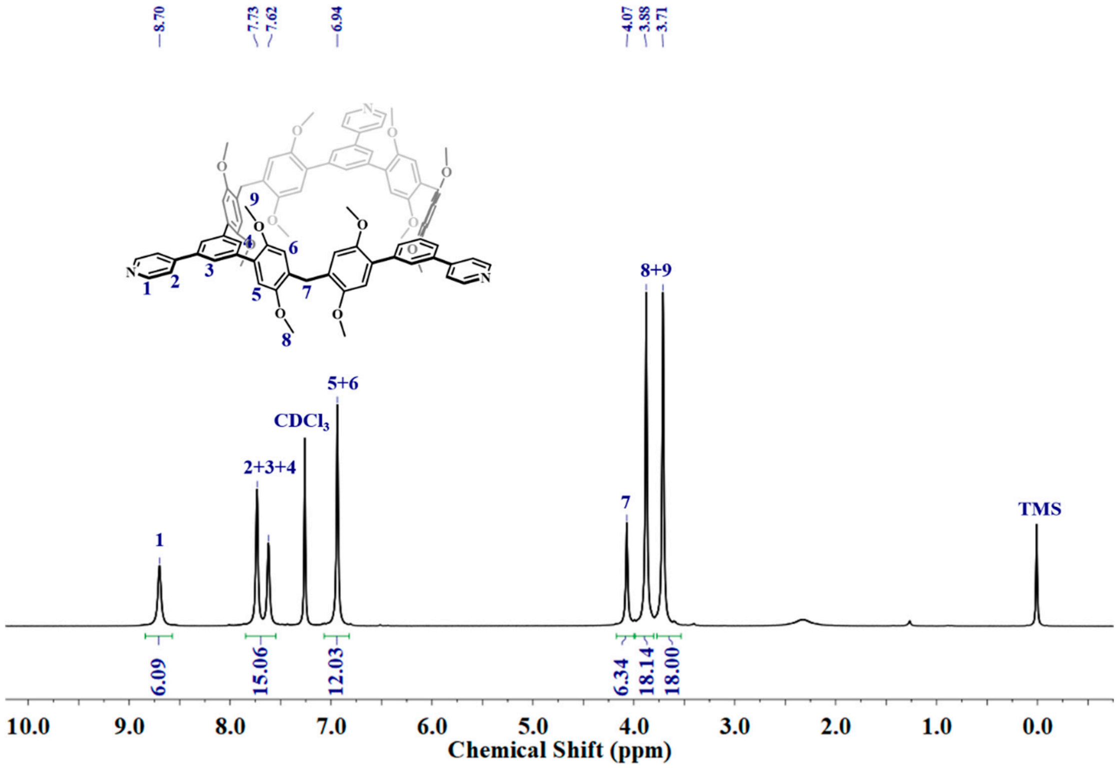

Figure S5. ^{1}H NMR spectrum (600 MHz, $CDCl_3$, 298 K) of **TP3**.

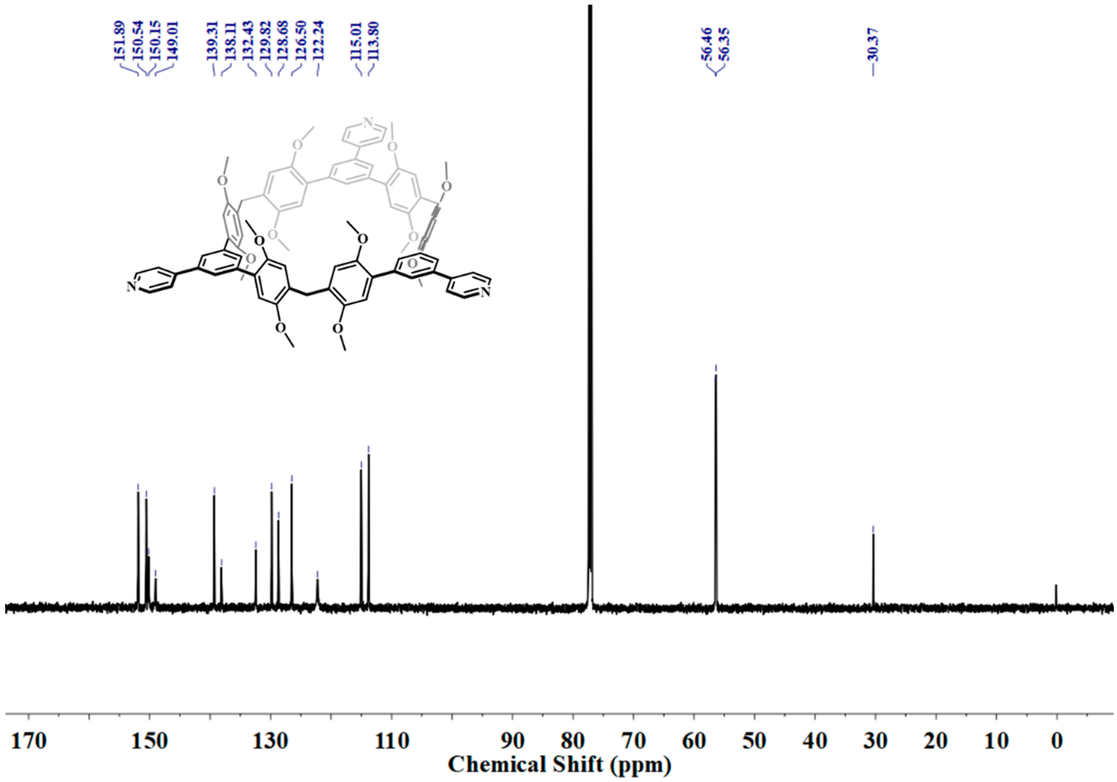

Figure S6. ^{13}C NMR spectrum (100 MHz, $CDCl_3$, 298 K) of **TP3-Br**.

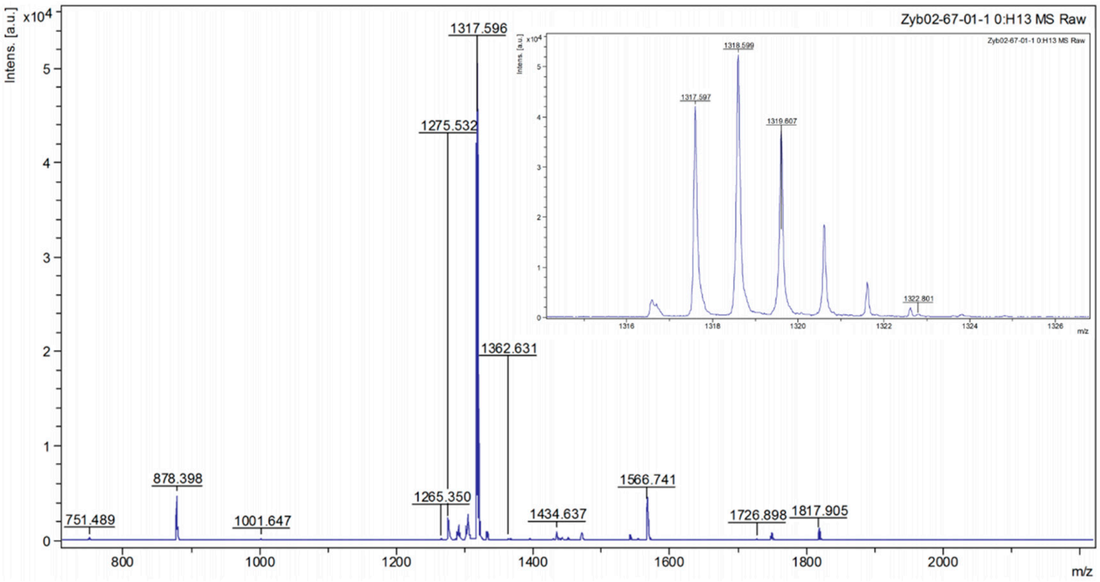

Figure S7. MALDI-TOF MS of **TP3-Br**.

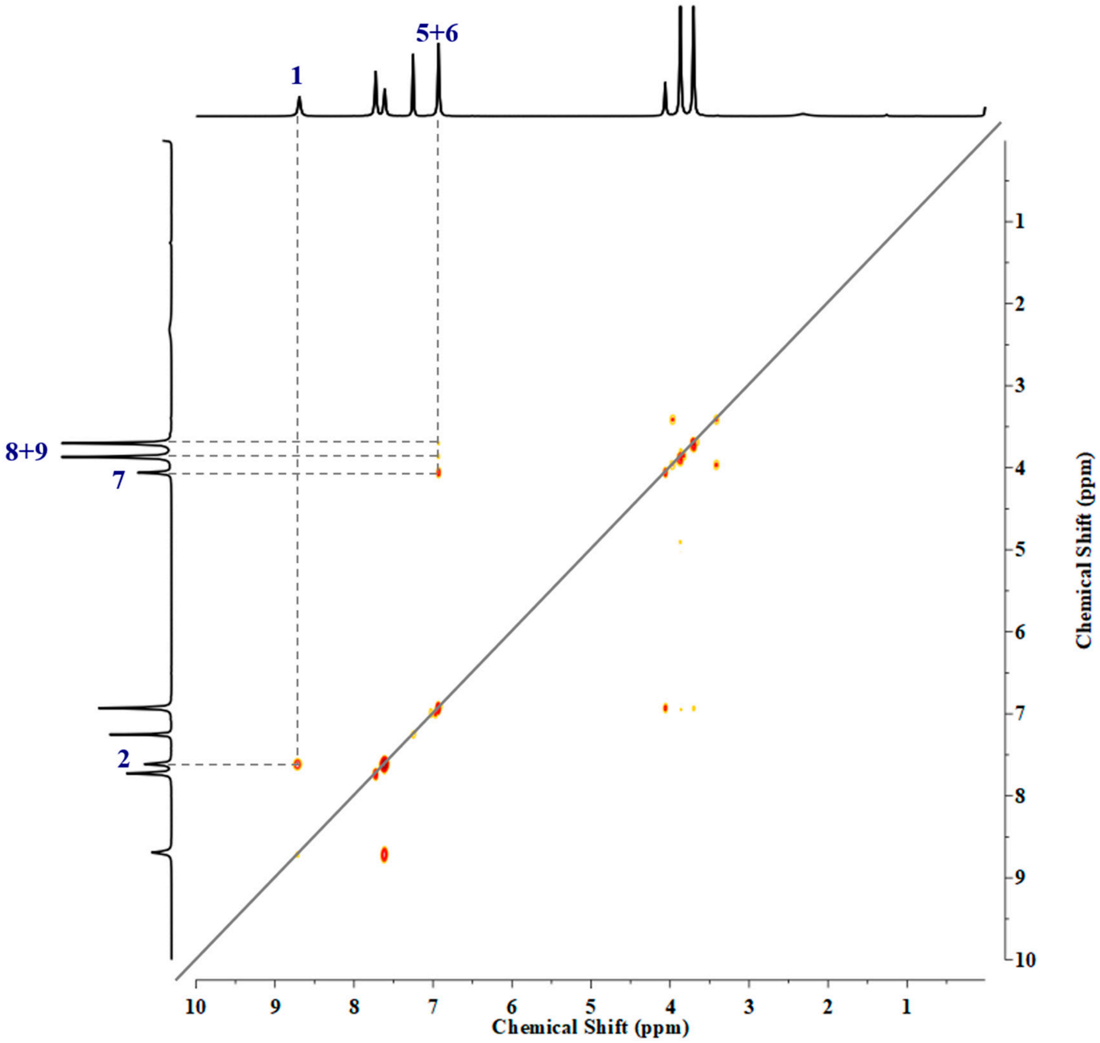

Figure S8. 2D COSY spectrum (400 MHz, $CDCl_3$, 298 K) of **TP3-Br**.

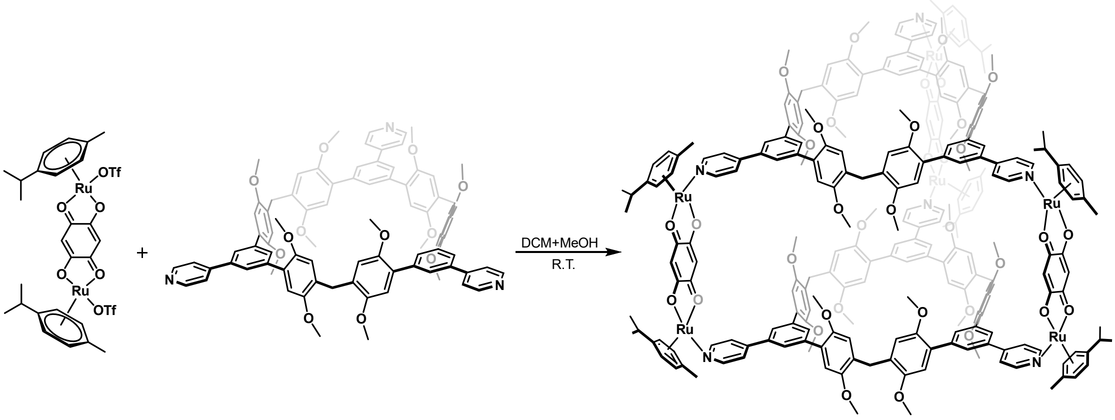

A1 TP3-Br M1

Scheme S1. Synthetic routes for the **M1**.

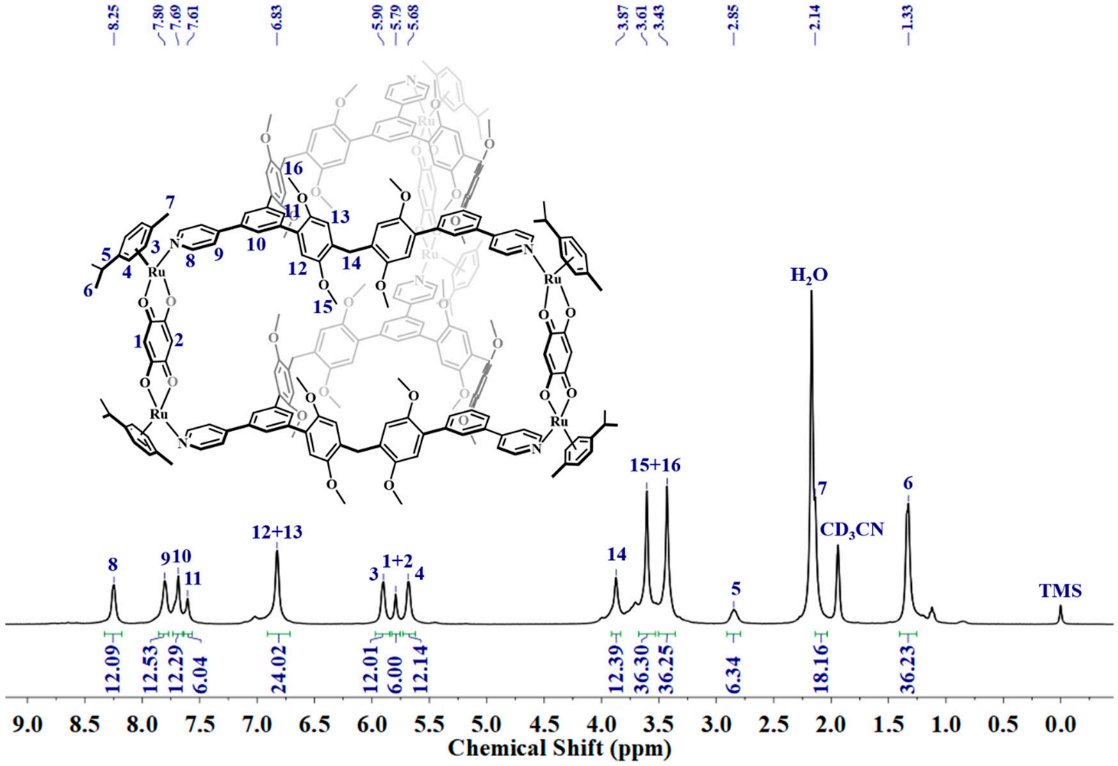

Figure S9. ^{1}H NMR spectrum (400 MHz, CD_3CN, 298 K) of **M1**.

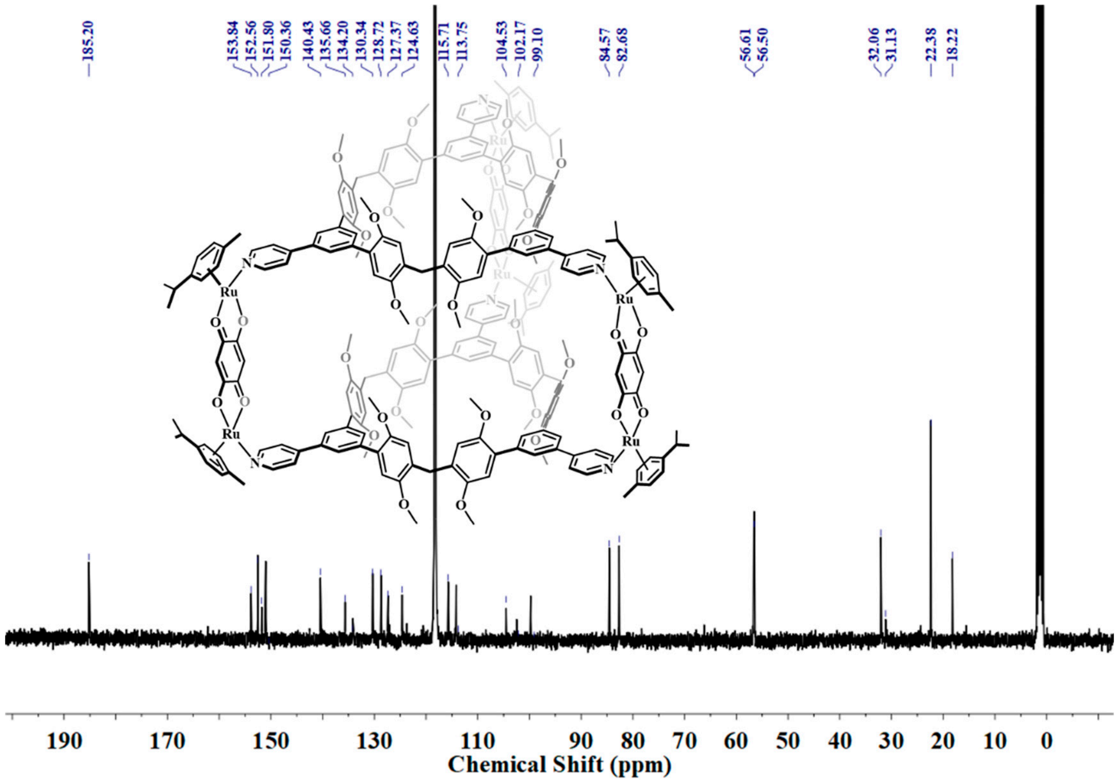

Figure S10. ^{13}C NMR spectrum (100 MHz, CD_3CN, 298 K) of **M1**.

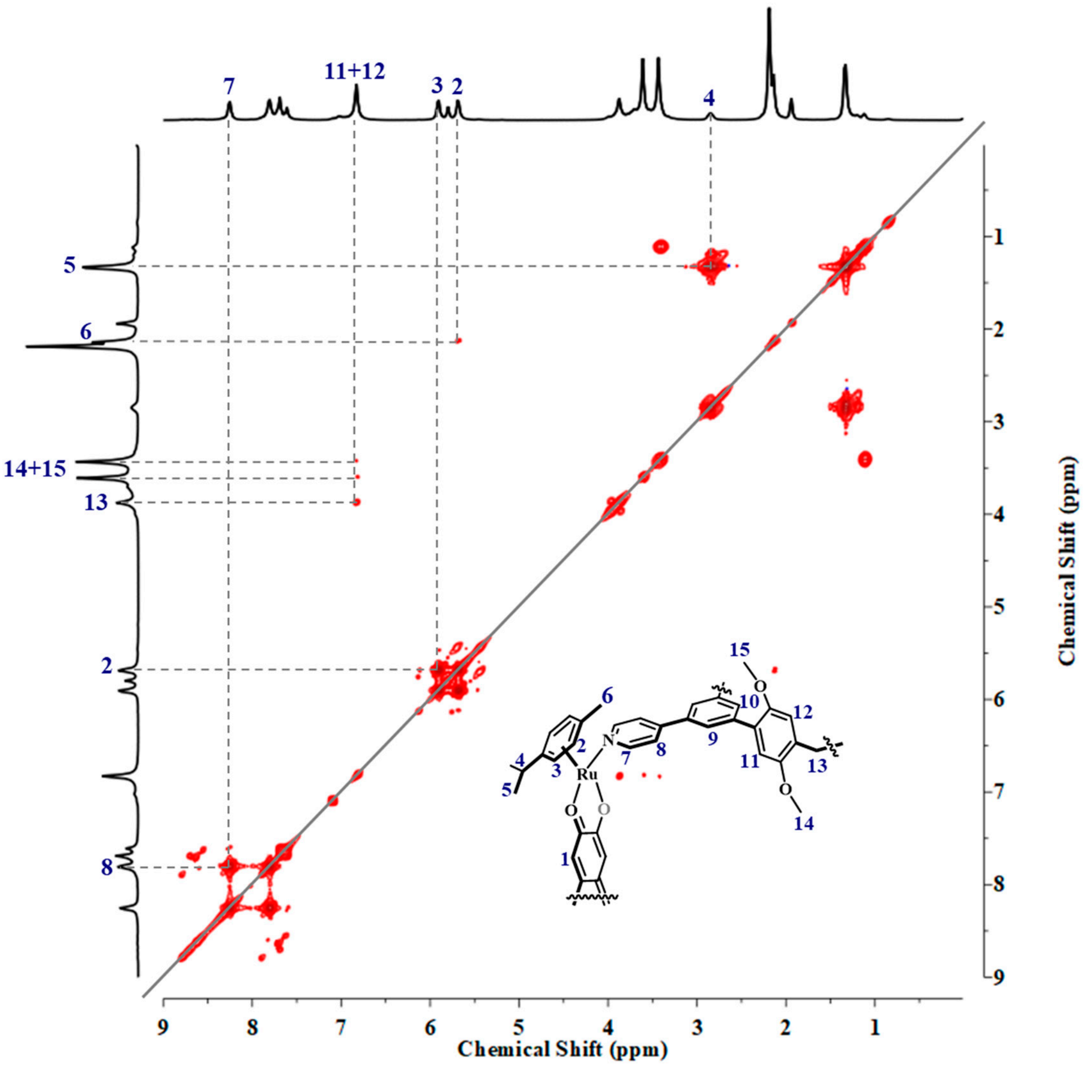

Figure S11. 2D COSY spectrum (400 MHz, CD_3CN, 298 K) of **M1**.

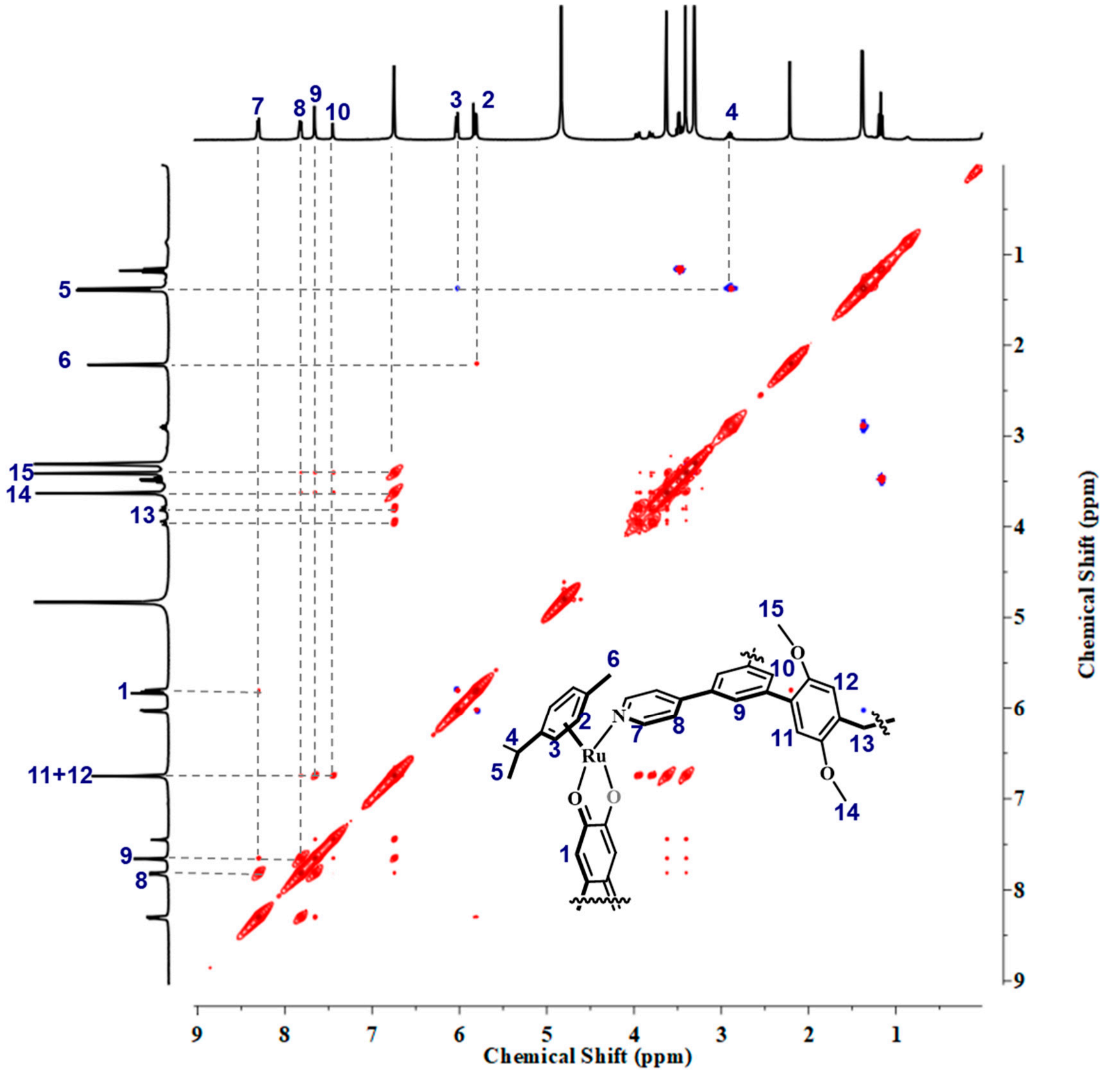

Figure S12. 2D NOESY spectrum (400 MHz, CD_3CN, 298 K) of **M1**.

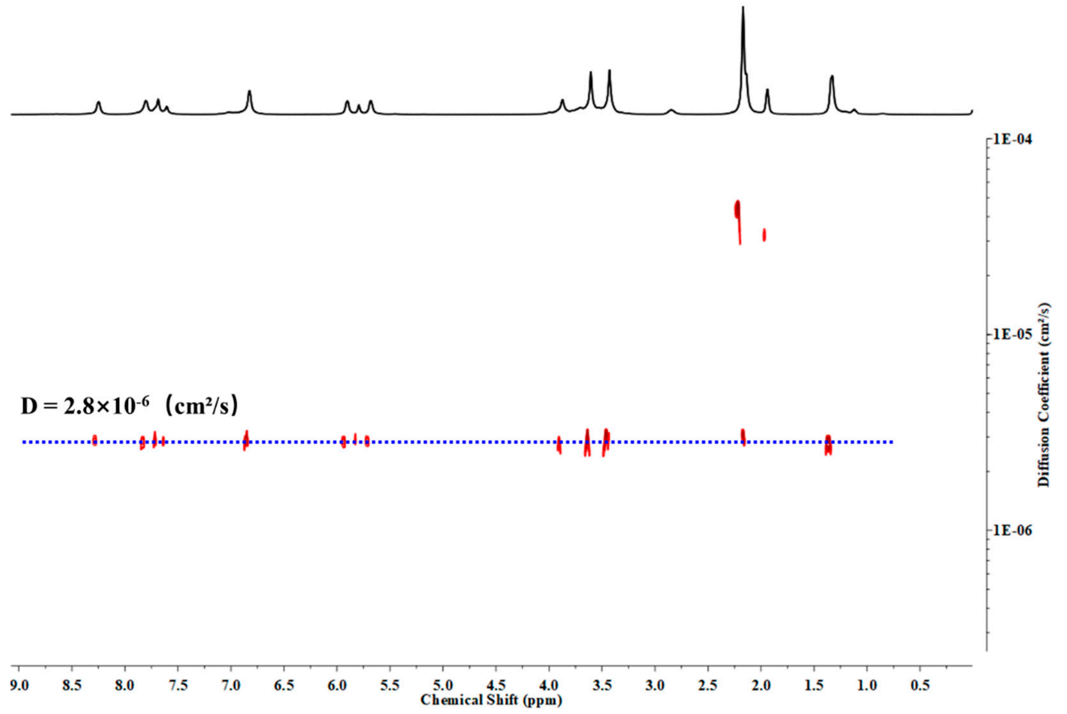

Figure S13. DOSY spectrum (400 MHz, CD_3CN, 298 K) of **M1**.

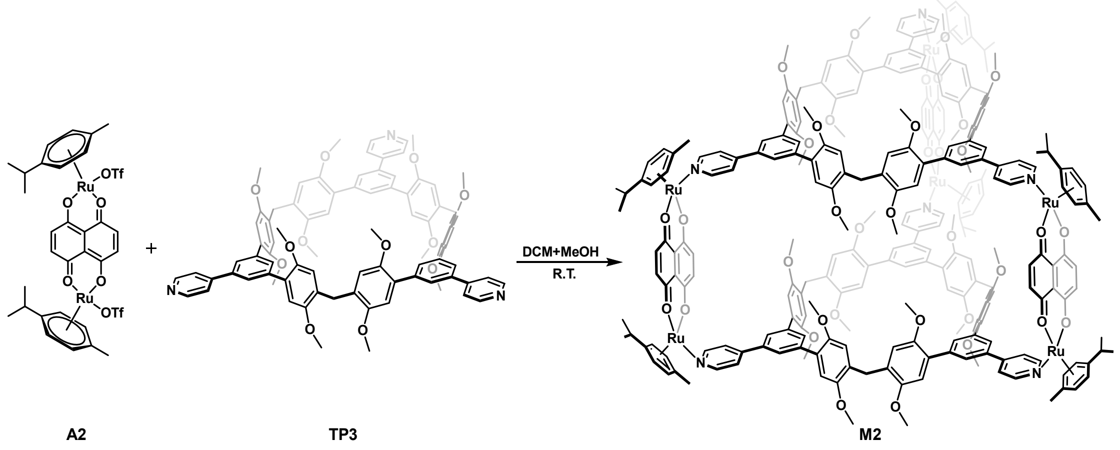

Scheme S2. Synthetic routes for the **M2**.

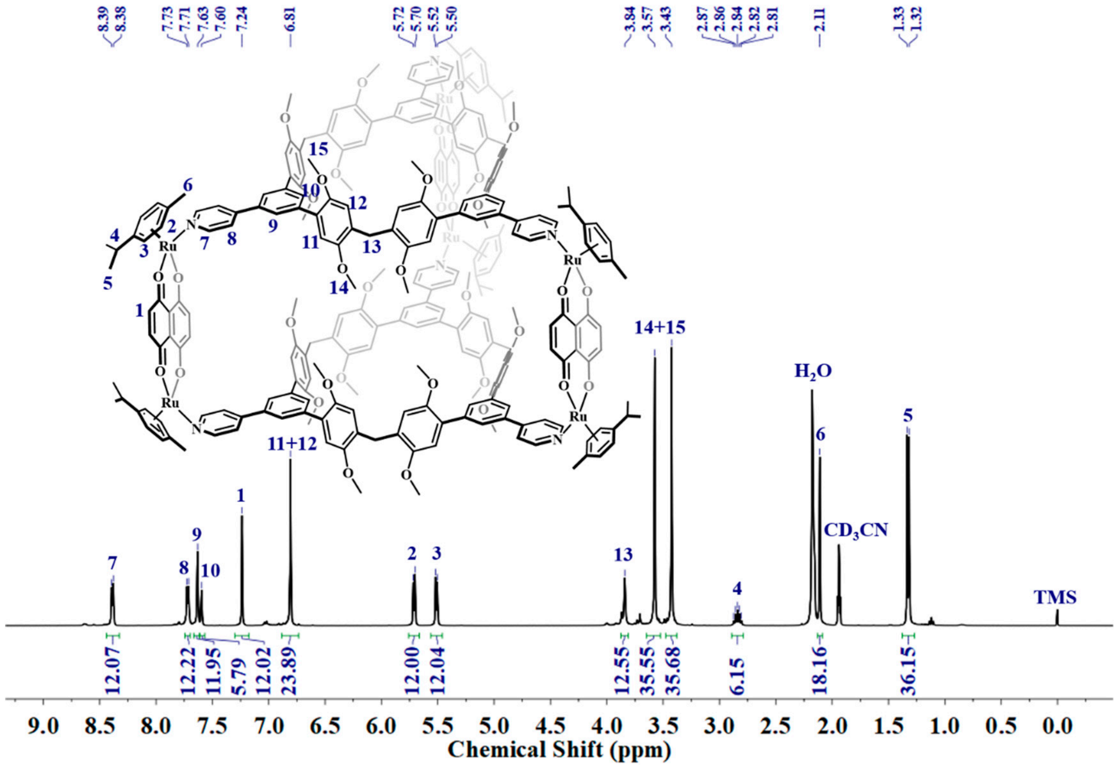

Figure S14. ^{1}H NMR spectrum (400 MHz, CD_3CN, 298 K) of **M2**.

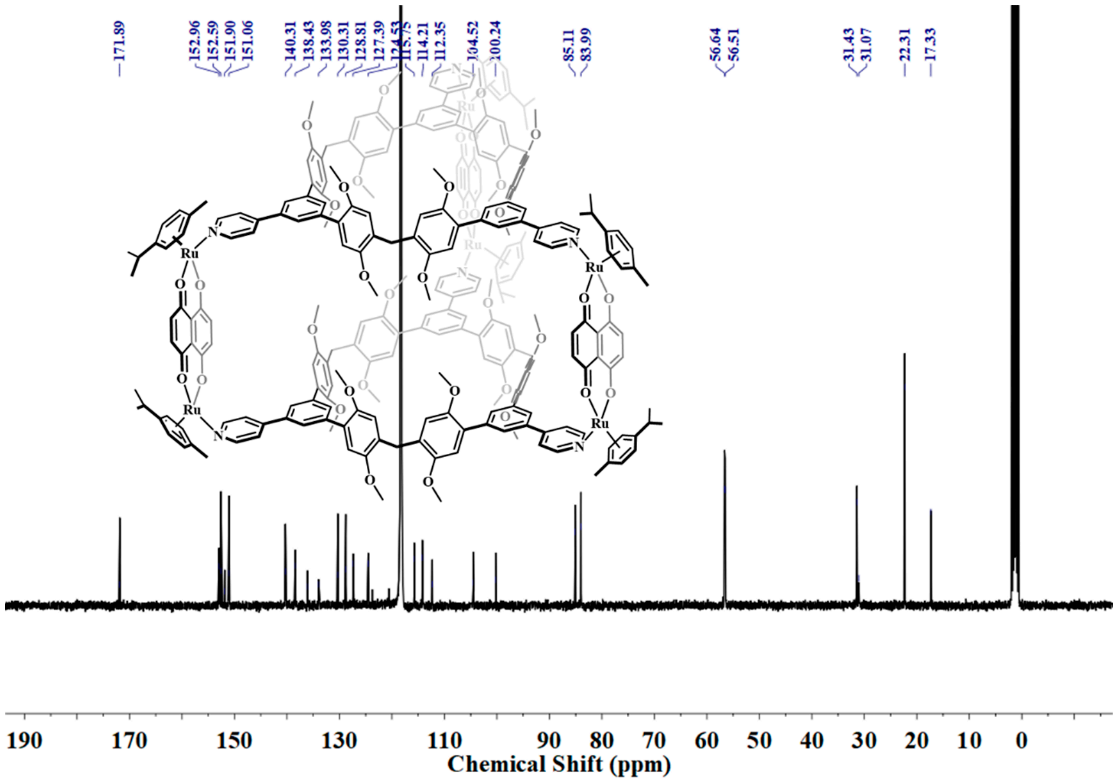

Figure S15. ^{13}C NMR spectrum (100 MHz, CD_3CN, 298 K) of **M2**.

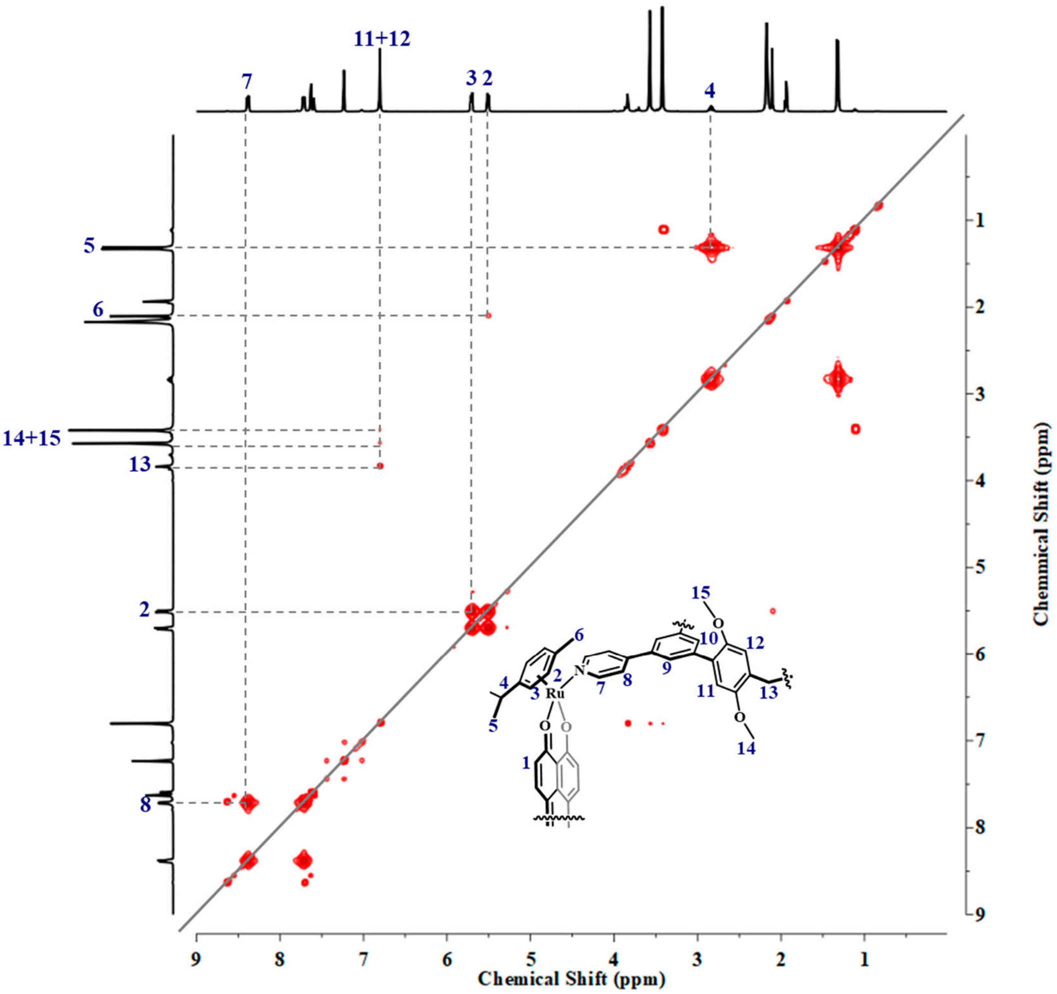

Figure S16. 2D COSY spectrum (400 MHz, CD_3CN, 298 K) of **M2**.

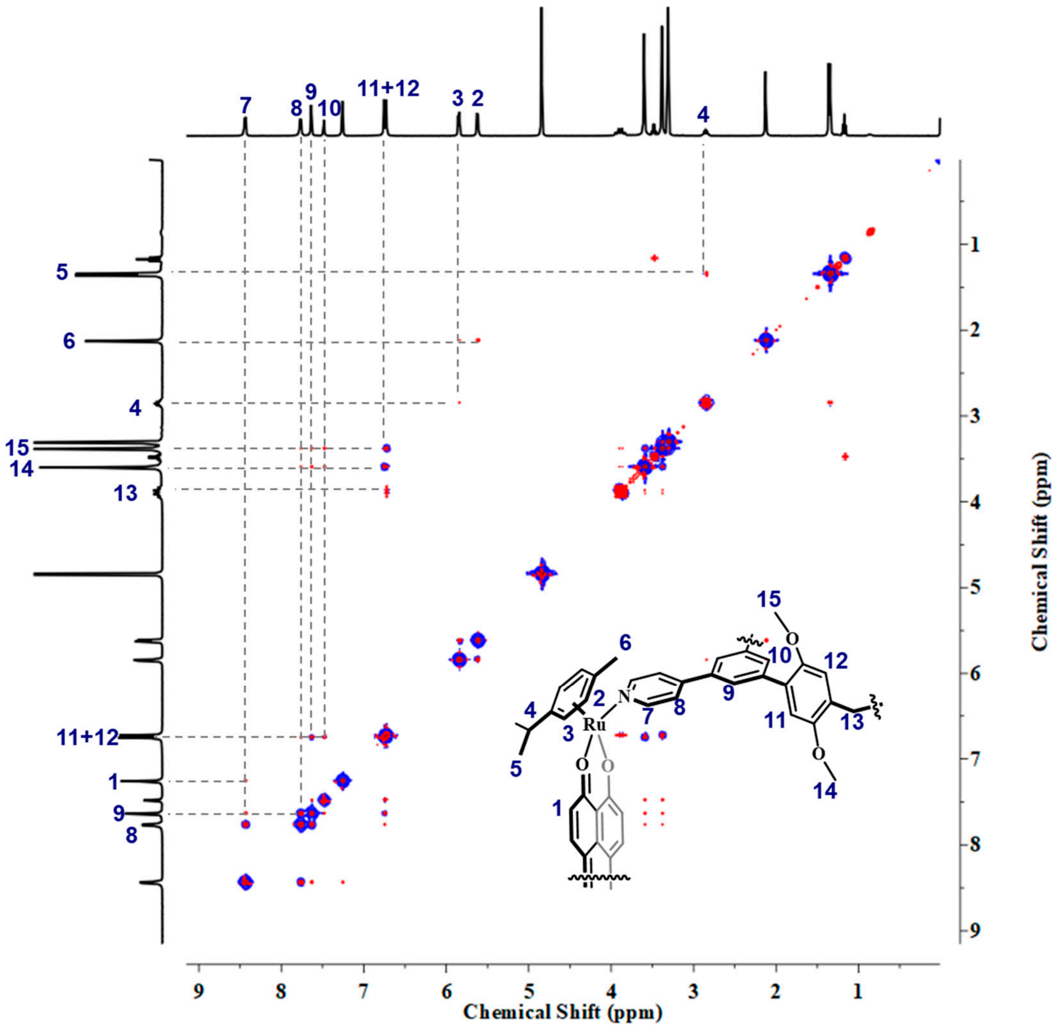

Figure S17. 2D NOESY spectrum (400 MHz, CD_3CN, 298 K) of **M2**.

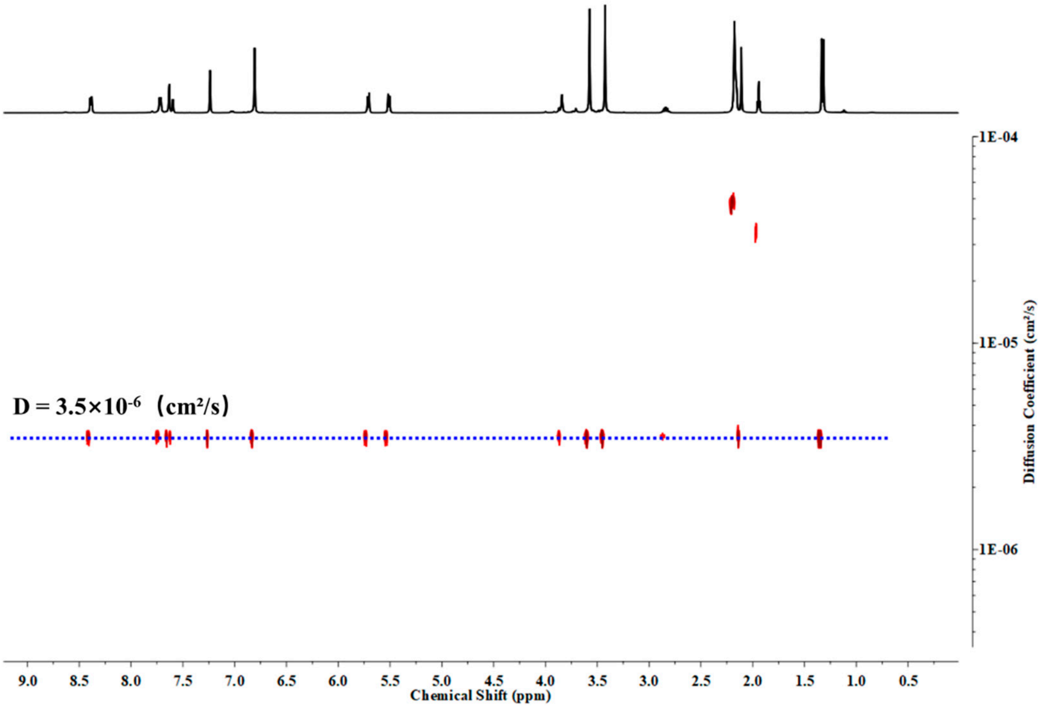

Figure S18. DOSY spectrum (400 MHz, CD_3CN, 298 K) of **M2**.

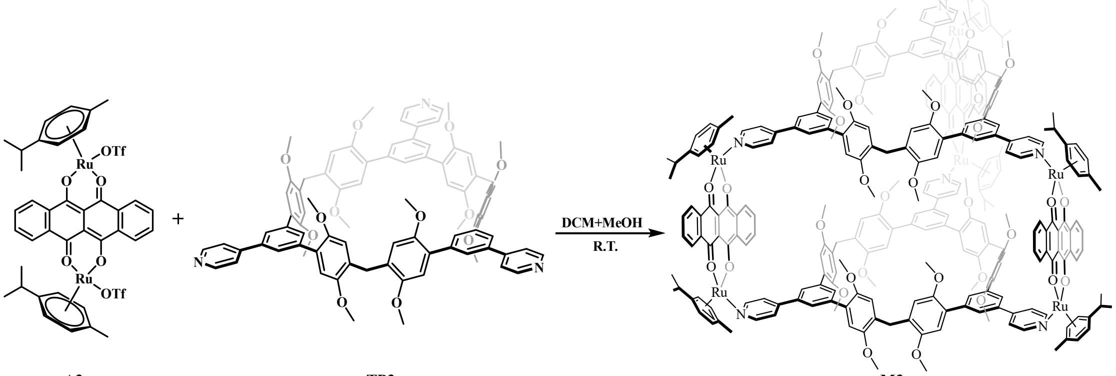

Scheme S3. Synthetic routes for the **M3**.

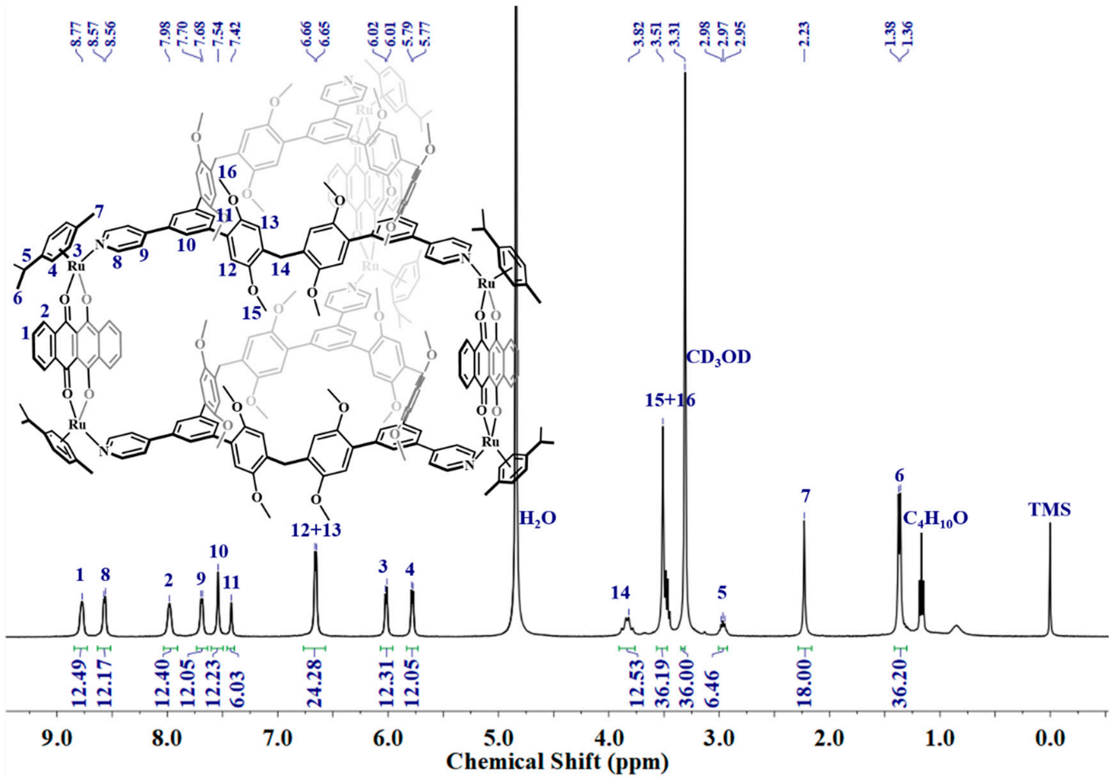

Figure S19. ^{1}H NMR spectrum (400 MHz, CD_3OD, 298 K) of **M3**.

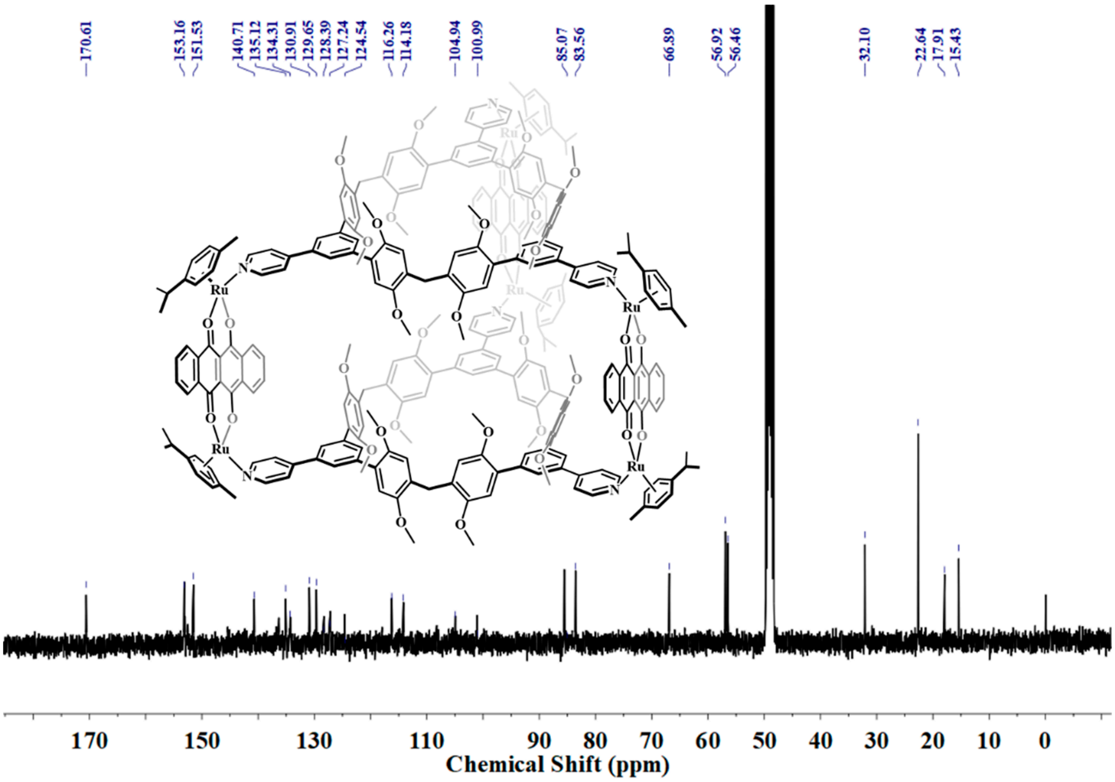

Figure S20. ^{13}C NMR spectrum (100 MHz, CD_3OD, 298 K) of **M3**.

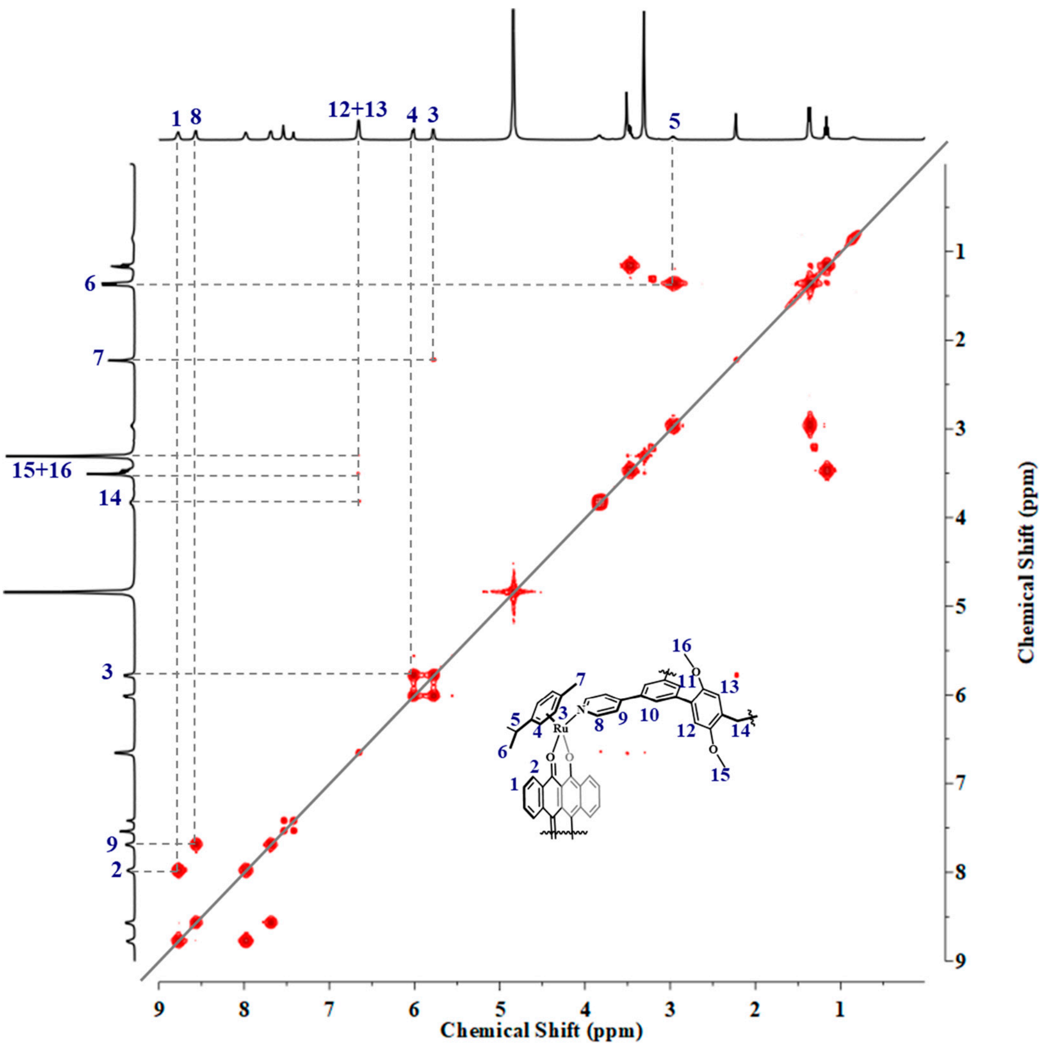

Figure S21. 2D COSY spectrum (400 MHz, CD_3OD, 298 K) of **M3**.

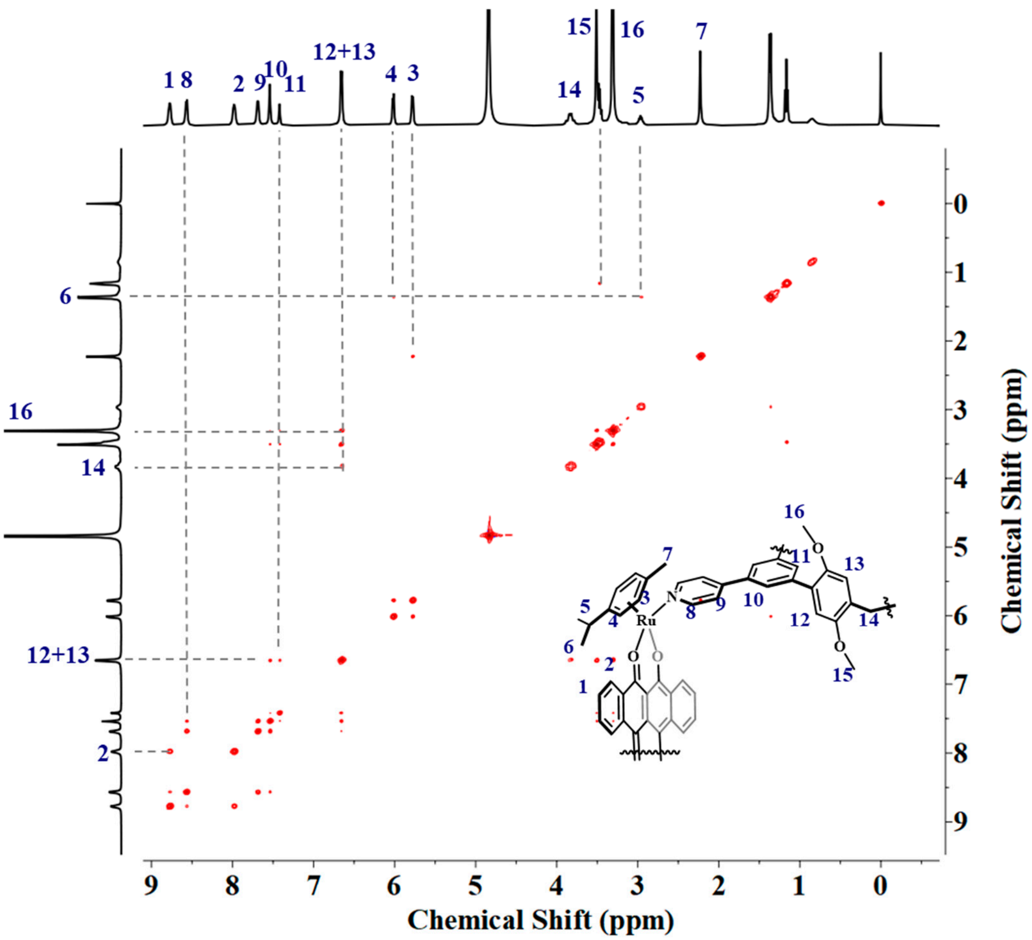

Figure S22. 2D NOESY spectrum (400 MHz, CD_3OD, 298 K) of **M3**.

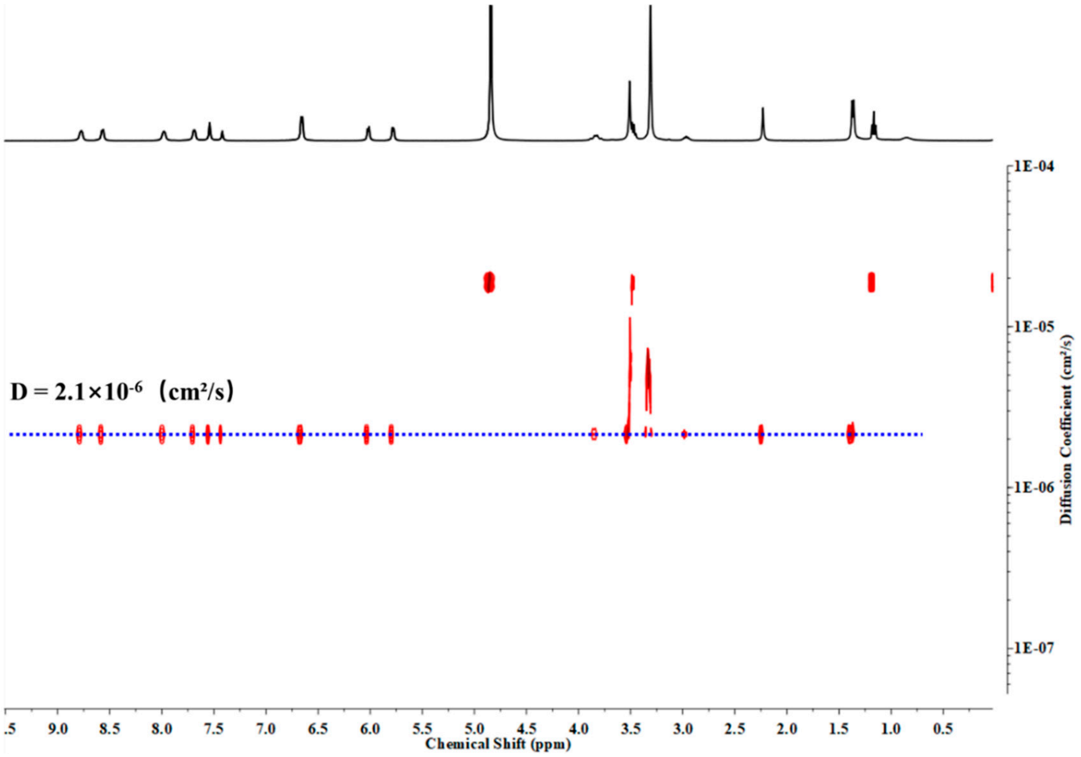

Figure S23. DOSY spectrum (400 MHz, CD_3CN, 298 K) of **M3**.

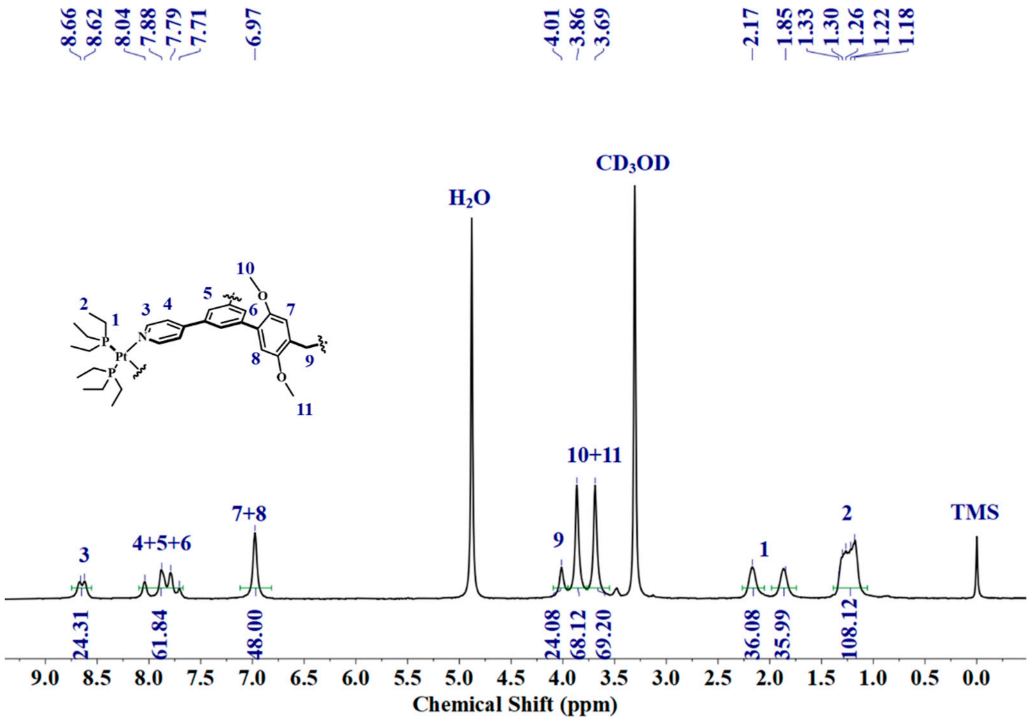

Figure S24. ^1H NMR spectrum (400 MHz, CD_3OD, 298 K) of **M4**.

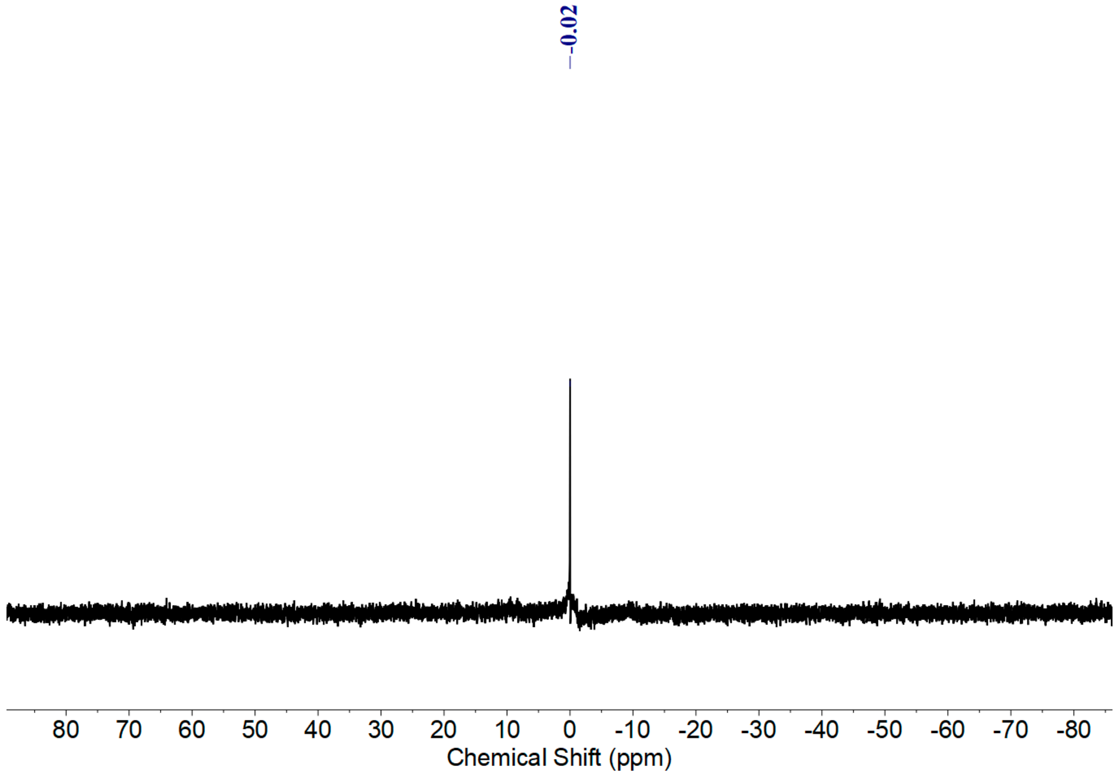

Figure S25. $^{31}P\{^1H\}$ NMR (162 MHz, CD_3OD, 25 °C) spectrum of **M4**.

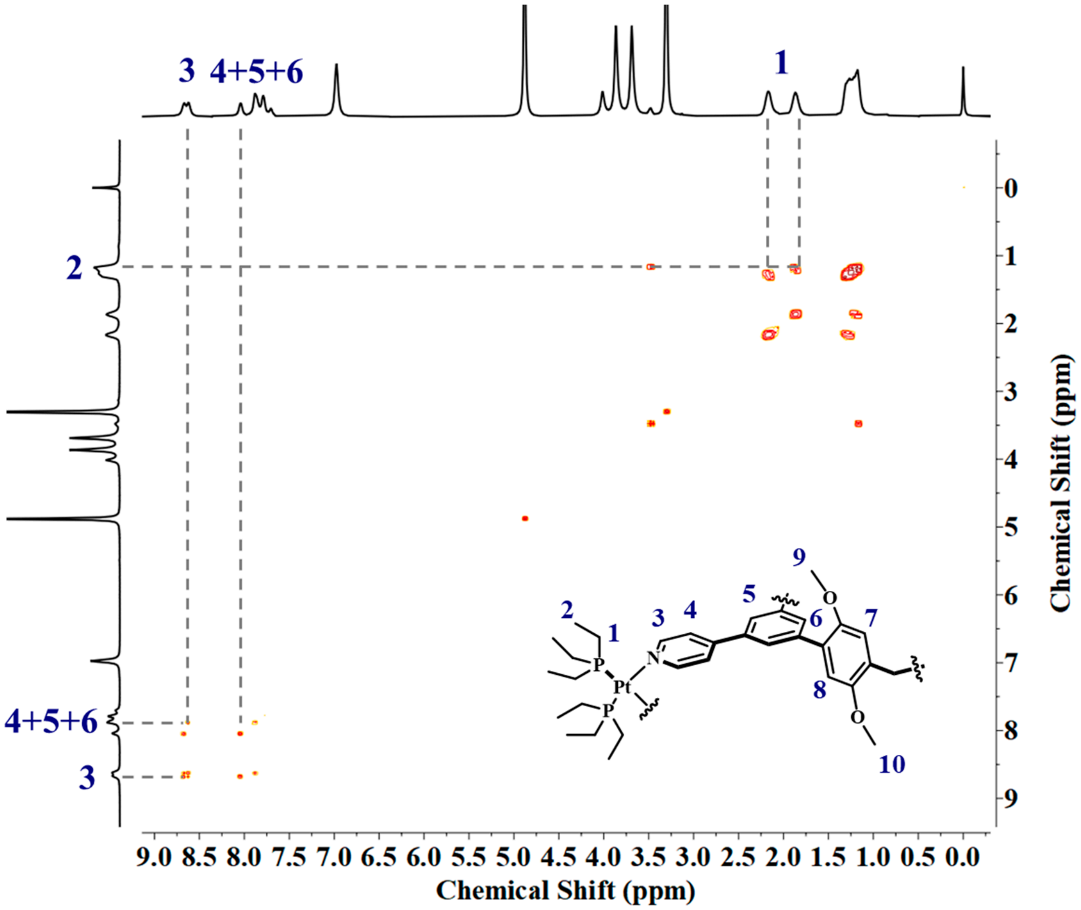

Figure S26. 2D COSY spectrum (400 MHz, CD_3OD, 298 K) of **M4**.

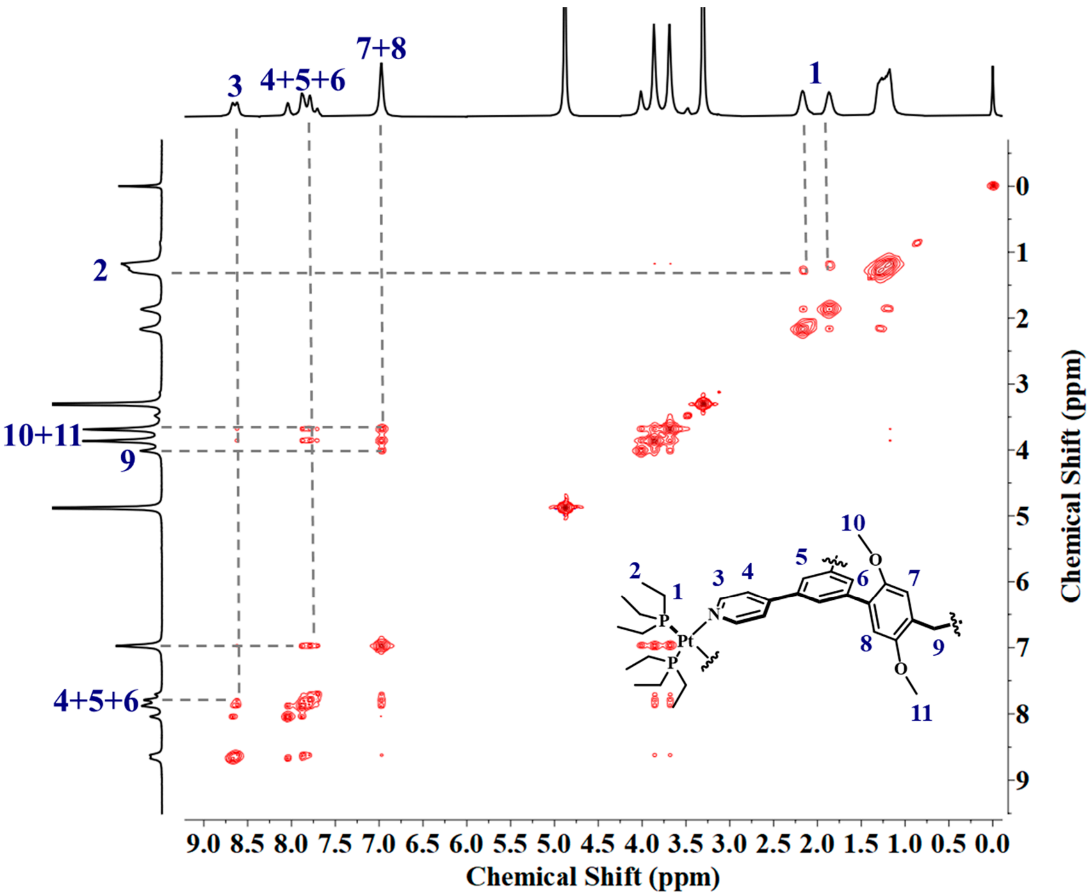

Figure S27. 2D NOESY spectrum (400 MHz, CD_3OD, 298 K) of **M4**.

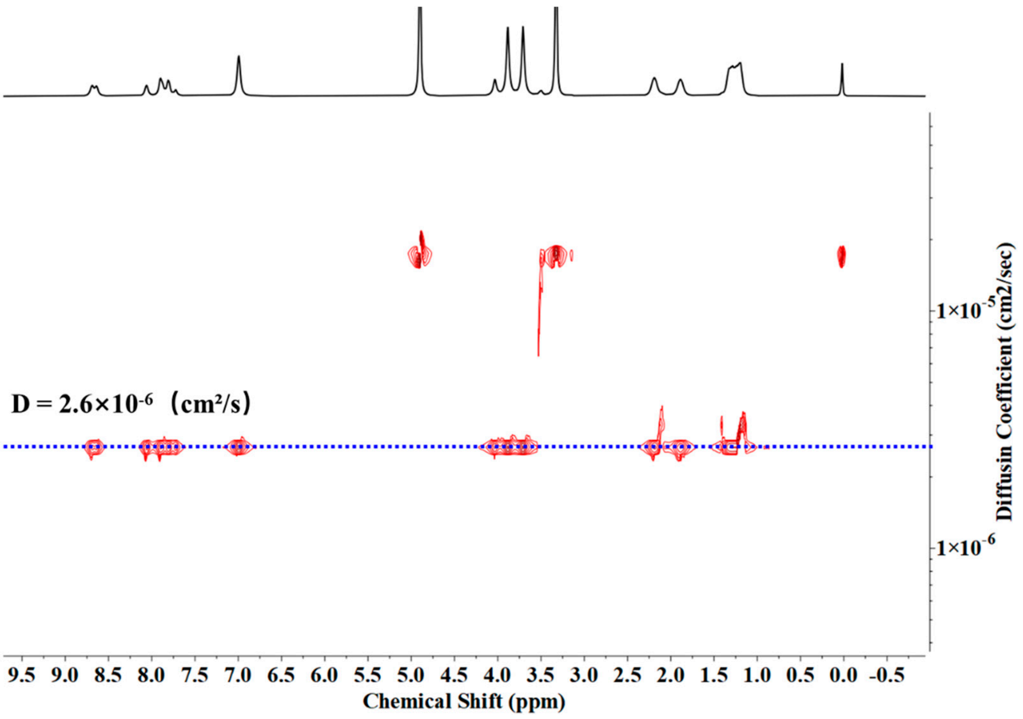

Figure S28. DOSY spectrum (400 MHz, CD_3OD, 298 K) of **M4**.

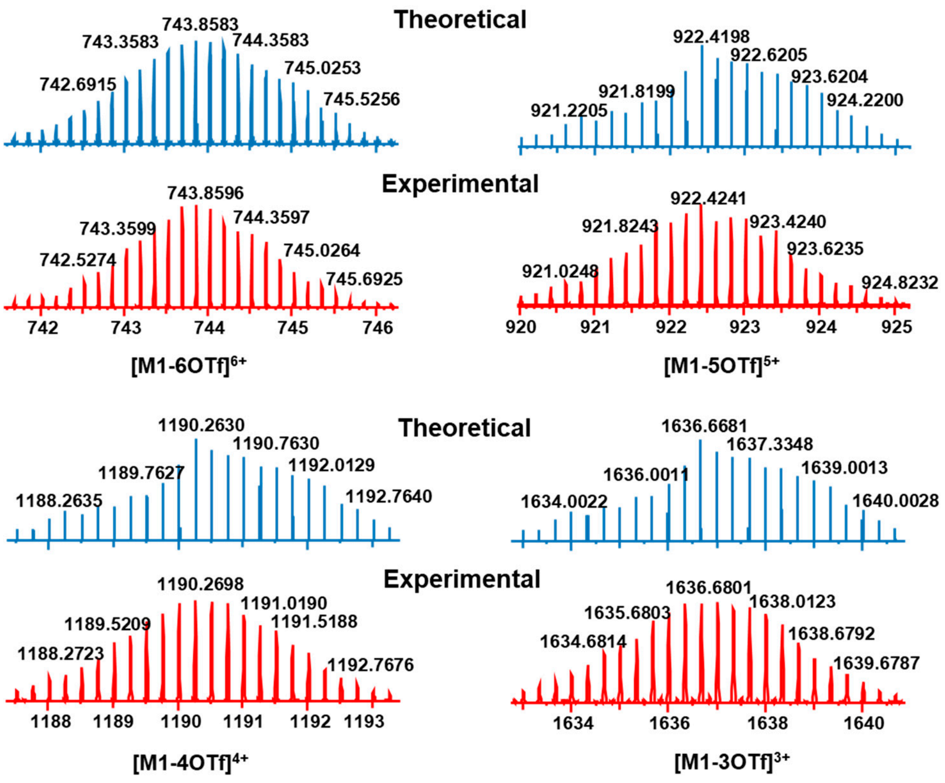

Figure S29. ESI-MS spectra of **M1**.

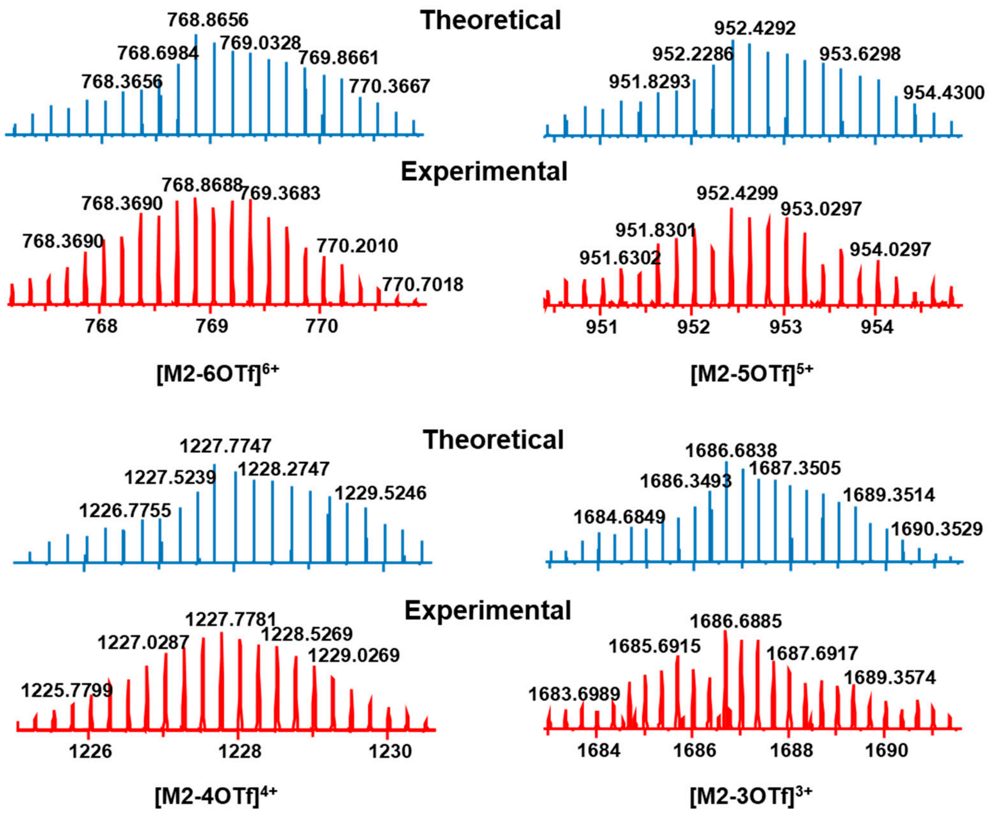

Figure S30. ESI-MS spectra of **M2**.

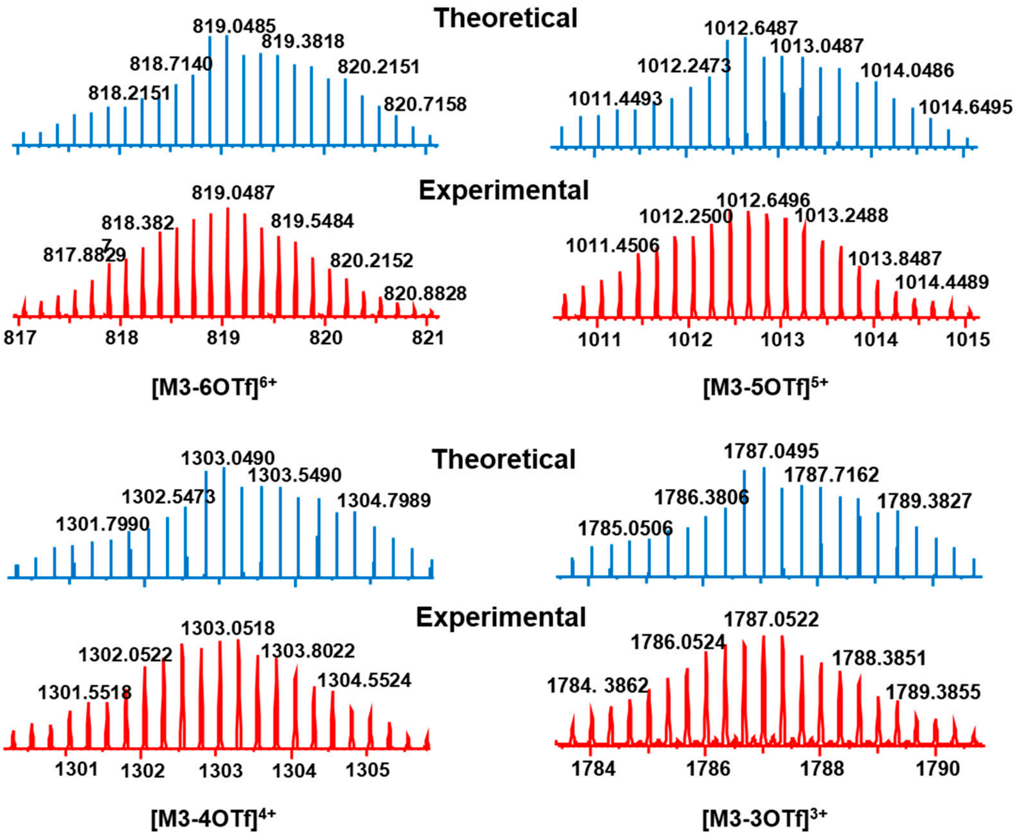

Figure S31. ESI-MS spectra of **M3**.

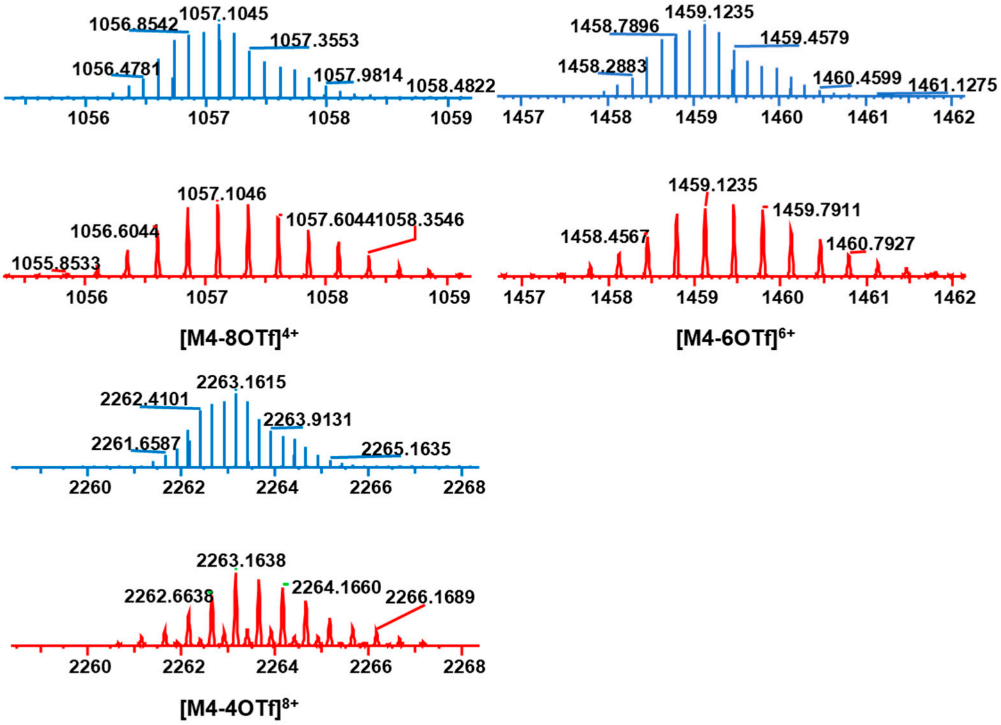

Figure S32. ESI-MS spectra of **M4**.

Section III. Single-crystal structures of TP3

Table S1. Crystal data of **TP3**.

Name	**TP3**
CCDC number	2238729
Empirical formula	$C_{84}H_{75}N_3O_{12}$
Formula weight	1318.47
Temperature / K	293(2)
Radiation / Å	CuKα (λ= 1.54184)
Crystal system	monoclinic
Space group	$P2_1/n$
a / Å	15.9766(11)
b / Å	25.8315(16)
c / Å	22.530(2)
α / °	90
β / °	104.916(2)
γ / °	90
Volume/ $Å^3$	8985.0(13)
Z	4
Density (calculated)	0.975 g/cm^3
μ / mm^{-1}	0.524
F(000)	2784.0
Crystal size (mm^3)	0.22×0.2×0.18
2θ range /°	5.308 to 134.16
Index ranges	$-12 \leq h \leq 19$
	$-29 \leq k \leq 30$
	$-26 \leq l \leq 21$
Reflections collected	34957
Independent reflections	15949
R(int)	0.0787
Data/restraints/parameters	15949/126/904
Goodness-of-fit on F^2	1.063
Final R indices [I>2sigma(I)]	R_1 = 0.1610
	wR_2 = 0.4034
R indices (all data)	R_1 = 0.2388
	wR_2 = 0.4969
Largest diff. peak/hole / e $Å^{-3}$	0.79/-0.40

Table S2. Crystal data of **M2**.

Name	**M2**
CCDC number	2238728
Empirical formula	$C_{288}H_{258}F_{18}N_6O_{54}Ru_6S_6$
Formula weight	5807.00
Temperature / K	99.98(13)
Radiation / Å	CuKα (λ= 1.54184)
Crystal system	monoclinic
Space group	I2/a
a / Å	40.6464(6)
b / Å	25.0517(3)
c / Å	34.0854(6)
α / °	90
β / °	108.460(2)
γ / °	90
Volume/ $Å^3$	32921.9(9)
Z	141
Density (calculated)	1.232 g/cm^3
μ / mm^{-1}	0.737
F(000)	12900.0
Crystal size (mm^3)	0.15×0.11×0.1
2θ range /°	4.462 to 149.622
Index ranges	$-48 \le h \le 50$
	$-30 \le k \le 26$
	$-41 \le l \le 42$
Reflections collected	96284
Independent reflections	32132
R(int)	0.0265
Data/restraints/parameters	32132/42/1555
Goodness-of-fit on F^2	2.401
Final R indices [I>2sigma(I)]	R_1 = 0.2146
	wR_2 = 0.5356
R indices (all data)	R_1 = 0.2375
	wR_2 = 0.5551
Largest diff. peak/hole / e $Å^{-3}$	6.25/-1.46

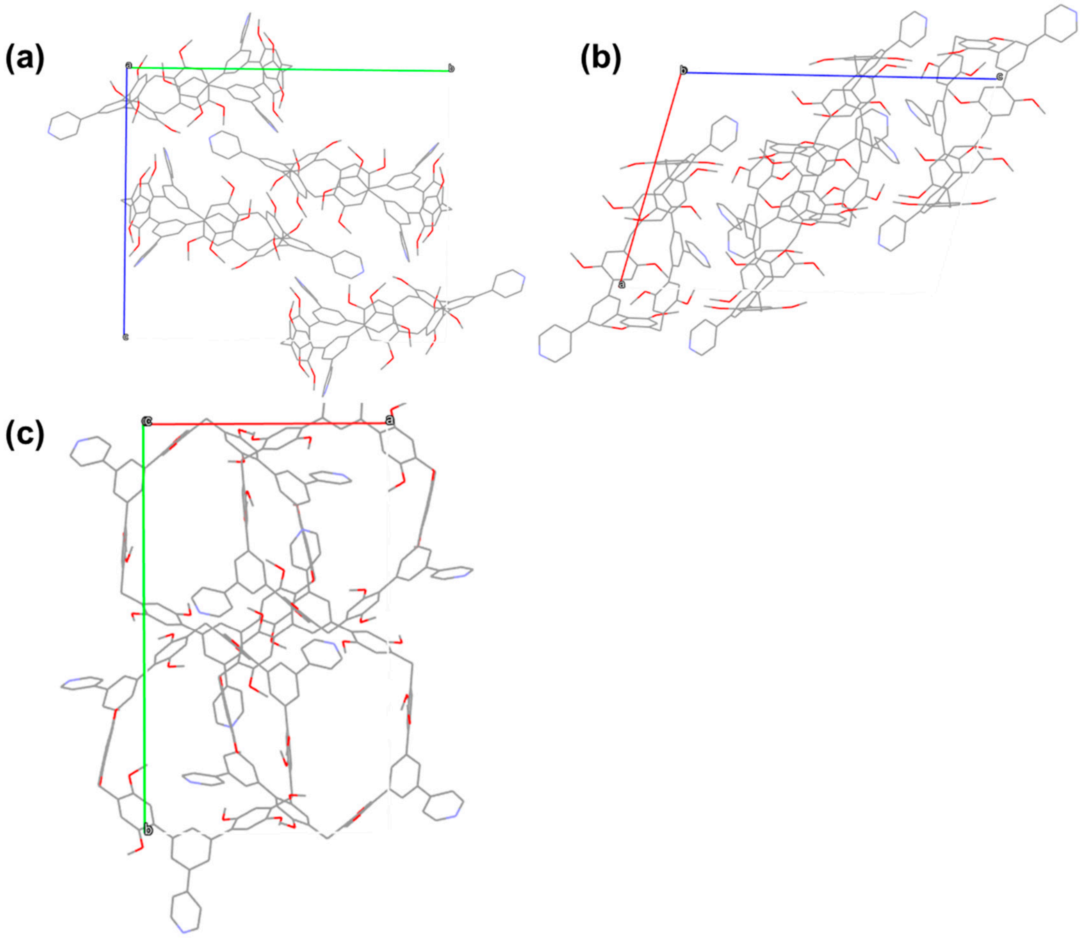

Figure S33. Packing diagram of **TP3** view along the (a) a-axis, (b) b-axis, and (c) c-axis.

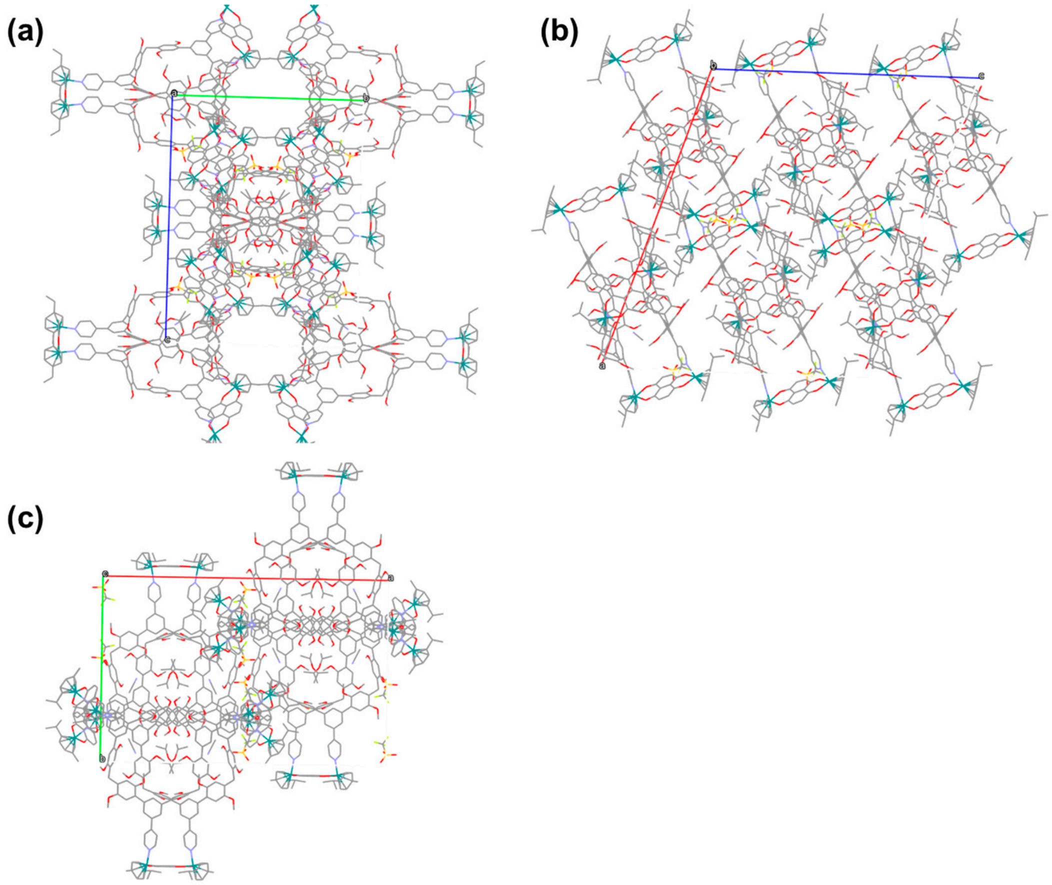

Figure S34. Packing diagram of **M2** view along the (a) a-axis, (b) b-axis, and (c) c-axis.
